# The contributions of DNA accessibility and transcription factor occupancy to enhancer activity during cellular differentiation

**DOI:** 10.1093/g3journal/jkad269

**Published:** 2023-11-22

**Authors:** Trevor Long, Tapas Bhattacharyya, Andrea Repele, Madison Naylor, Sunil Nooti, Shawn Krueger, Manu

**Affiliations:** Department of Biology, University of North Dakota, Grand Forks, ND 58202-9019, USA; Department of Biology, University of North Dakota, Grand Forks, ND 58202-9019, USA; Department of Biology, University of North Dakota, Grand Forks, ND 58202-9019, USA; Department of Biology, University of North Dakota, Grand Forks, ND 58202-9019, USA; Department of Biology, University of North Dakota, Grand Forks, ND 58202-9019, USA; Department of Biology, University of North Dakota, Grand Forks, ND 58202-9019, USA; Department of Biology, University of North Dakota, Grand Forks, ND 58202-9019, USA

**Keywords:** ATAC-Seq, Luciferase assay, enhancer3, gene regulation, transcription factor, chromatin dynamics

## Abstract

During gene regulation, DNA accessibility is thought to limit the availability of transcription factor (TF) binding sites, while TFs can increase DNA accessibility to recruit additional factors that upregulate gene expression. Given this interplay, the causative regulatory events in the modulation of gene expression remain unknown for the vast majority of genes. We utilized deeply sequenced ATAC-Seq data and site-specific knock-in reporter genes to investigate the relationship between the binding-site resolution dynamics of DNA accessibility and the expression dynamics of the enhancers of *Cebpa* during macrophage-neutrophil differentiation. While the enhancers upregulate reporter expression during the earliest stages of differentiation, there is little corresponding increase in their total accessibility. Conversely, total accessibility peaks during the last stages of differentiation without any increase in enhancer activity. The accessibility of positions neighboring C/EBP-family TF binding sites, which indicates TF occupancy, does increase significantly during early differentiation, showing that the early upregulation of enhancer activity is driven by TF binding. These results imply that a generalized increase in DNA accessibility is not sufficient, and binding by enhancer-specific TFs is necessary, for the upregulation of gene expression. Additionally, high-coverage ATAC-Seq combined with time-series expression data can infer the sequence of regulatory events at binding-site resolution.

## Introduction

Metazoan gene expression is regulated intricately in time during cellular differentiation (Spitz and Furlong 2012; May *et al.* 2013; Choi *et al.* 2021). The cell-type specific temporal pattern of a gene’s expression is encoded in DNA as clusters of transcription factor (TF) binding sites called *cis*-regulatory modules (CRMs) or enhancers (Mouse ENCODE Consortium *et al.* 2012; Spitz and Furlong 2012). TFs bound to enhancers recruit coactivators or corepressors that interact with the RNA polymerase holoenzyme complex directly or through the Mediator complex (Kagey *et al.* 2010; Boija *et al.* 2018). In addition to the action of sequence-specific TFs, the accessibility of enhancer DNA, which is inversely related to nucleosome occupancy, has been linked to enhancers’ ability to drive higher levels of gene expression (Kornberg and Lorch 1992; Bartsch *et al.* 1996; Mellor 2005; Boyle *et al.* 2008; Thurman *et al.* 2012; Buenrostro *et al.* 2013). While enhancer accessibility is thought to influence the occupancy of sequence-specific TFs, bound TFs also regulate accessibility by either actively recruiting chromatin remodeling enzymes, or passively by competing with nucleosomes (Adams and Workman 1995; Boyes and Felsenfeld 1996; Mellor 2005; Ptashne 2013). Furthermore, enhancers, especially those regulating developmental genes, have complex regulation and some are regulated by 6–8 TFs binding to multiple sites for each TF (Levine and Davidson 2005; Kim *et al.* 2013; Cooper *et al.* 2015; Farley *et al.* 2015; Repele *et al.* 2019; Kim *et al.* 2021). Consequently, the causative regulatory events underlying the modulation of gene expression in time remain unknown for the vast majority of genes.

*CCAAT/Enhancer binding protein, *α** (*Cebpa*) encodes a TF that is necessary for neutrophil development (Zhang *et al.* 1997) as well as the specification of hepatocytes and adipocytes (Legraverend *et al.* 1993; Rosen *et al.* 2002). During hematopoiesis, Cebpa is expressed in hematopoietic stem cells, granulocyte-monocyte progenitors (GMPs), neutrophils, and macrophages (http://biogps.org/gene/12606; Lattin *et al.* 2008; Wu *et al.* 2016). Although the most apparent hematopoietic phenotype of Cebpa ^−/−^ mice is neutropenia Zhang *et al.* (1997), *Cebpa* also has a role in specifying macrophages. *Cebpa* is expressed at intermediate and high levels in macrophages and neutrophils, respectively and the cell-fate decision is thought to depend on the ratio of PU.1, a TF necessary for all white-blood cell lineages Scott *et al.* (1994), and C/EBP*α* expression levels Dahl *et al.* (2003).

**Fig. 1.**
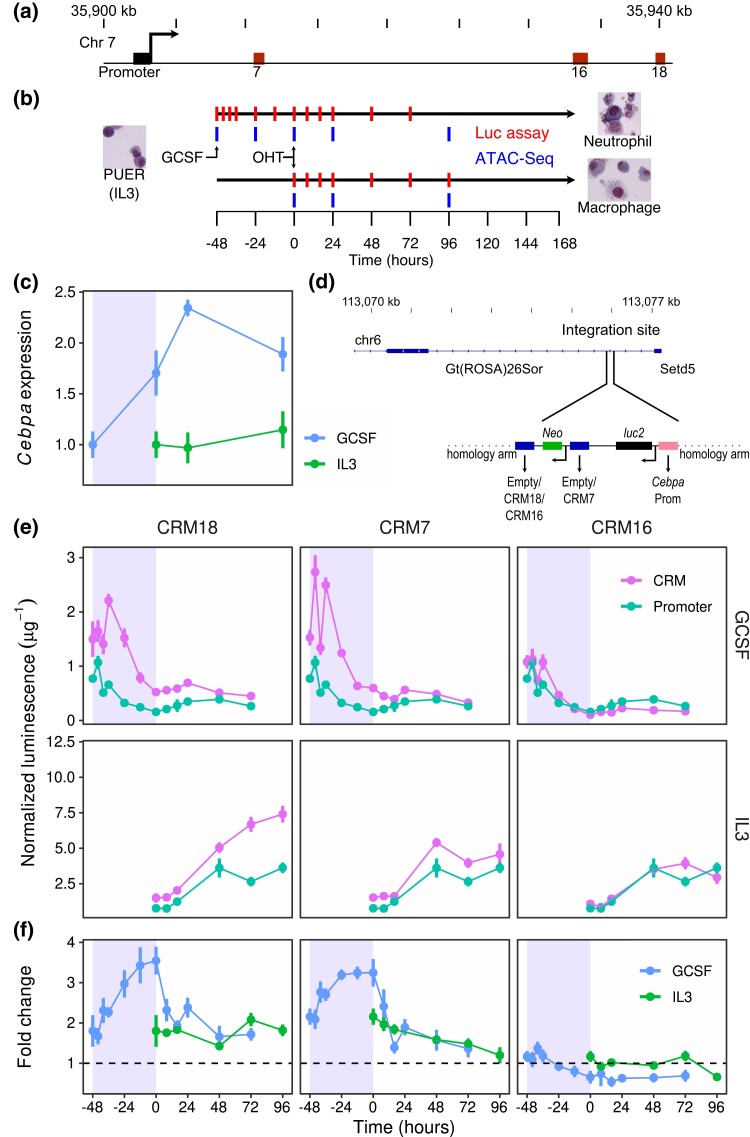
Expression driven by *Cebpa* CRMs during PUER cell differentiation. a) A 40 kb region of chromosome 7 showing the *Cebpa* transcription start site (TSS), promoter, and CRMs (enhancers) 7, 16, and 18. b) PUER cells may be differentiated into macrophages over a 7-day period by inducing with OHT in IL3 medium. Substituting GCSF for IL3 48 h prior to OHT induction results in neutrophil differentiation. The tick marks indicate timepoints at which Luciferase assays and ATAC-Seq were conducted. c) The time course of *Cebpa* expression relative to *hprt* measured by RT-qPCR during PUER differentiation. The shaded region shows period of GCSF pretreatment prior to induction with OHT during neutrophil differentiation. d) Site-specific knock in of Luciferase reporters into intron 1 of the ROSA26 locus by CRISPR/Cas9 HDR. *luc2* gene expression was driven by the *Cebpa* promoter (orange) alone or along with CRM 7, 16, or 18. e) Time series of Luciferase expression normalized to total protein measured in μg of BSA. Expression in GCSF and IL3 conditions is shown in the top and bottom panels, respectively. Promoter is the construct bearing only the *Cebpa* promoter, whereas CRM is the construct bearing CRM 18, 7, or 16 along with the *Cebpa* promoter in the left, middle, or right panels respectively. f) Fold change in the expression of enhancer-bearing reporters relative to the promoter at each time point.

Reflecting it’s functions in multiple tissues, *Cebpa* has a complex gene regulatory architecture. The promoter is bound by C/EBP family, Usf1, NF-Y family, Myc, ZNF143, and two other unknown TFs (Legraverend *et al.* 1993; Gonzalez *et al.* 2017). In addition to the promoter, *Cebpa* is regulated by three enhancers located 8, 31, and 37 kb downstream of the *Cebpa* transcription start site (TSS) in the mouse genome (Fig. 1a). The enhancer located at 37 kb is bound by PU.1, other Ets TFs, C/EBP, Runx1, SCL, Gata2, and Myb (Cooper *et al.* 2015; Bertolino *et al.* 2016; Guo *et al.* 2016; Repele *et al.* 2019) in myeloid cells. The enhancer at 8 kb is bound by C/EBP and Gfi1 (Bertolino *et al.* 2016; Peng *et al.* 2019; Repele *et al.* 2019) and the third enhancer is bound by PU.1 (Bertolino *et al.* 2016; Repele *et al.* 2019). While the binding sites and identities of the TFs regulating *Cebpa* enhancers have been established, it is not understood how the regulatory contributions of these TFs modulate *Cebpa*’s gene expression during myeloid differentiation. In particular, neither the dynamics of enhancer-driven expression, nor the dynamics of enhancer chromatin state have been characterized.

We profiled DNA accessibility at binding-site resolution as well as the dynamics of both accessibility and gene expression of the *Cebpa* enhancers during the differentiation of an *in vitro* model of myeloid development. PU.1^−/−^ cells from mouse bone marrow carrying an *Spi1* (PU.1) transgene fused to the ligand-binding domain of the estrogen receptor (PUER; Walsh *et al.* 2002) can be inducibly differentiated into macrophages or neutrophils in IL3 or GCSF, respectively (Fig. 1b; Dahl *et al.* 2003; Laslo *et al.* 2006; Bertolino *et al.* 2016; Repele *et al.* 2019). *Cebpa* gene expression is upregulated by GCSF treatment during neutrophil differentiation but not during macrophage differentiation (Fig. 1c; Dahl *et al.* 2003; Repele *et al.* 2019). In order to measure the upregulation driven by each enhancer at a high temporal resolution, we used CRISPR/Cas9 to knock in Luciferase reporters containing the *Cebpa* promoter either alone or together with one of the enhancers, CRM 7 (+8 kb), 16 (+31 kb), or 18 (+37 kb), in the same site in the ROSA26 locus in a biallelic manner (Fig. 1d). We measured the activity of each enhancer as the fold change in gene expression relative to the promoter, both in bulk and at the single-cell level using a flow cytometry-based method we developed for the purpose.

The temporal pattern of the fold change of CRMs 7 and 18 matches that of the endogenous gene, showing that they are primarily responsible for the GCSF-dependent upregulation during neutrophil differentiation. Pooling ATAC-Seq samples allowed us to achieve an average depth of coverage of ∼50 Tn5 cuts per nucleotide in the accessible regions of the genome, furnishing a very high resolution picture of intraenhancer accessibility. These high resolution profiles contained footprints that match all previously characterized binding sites and overlap ChIP signal peaks of the cognate TFs. While enhancer activity and total accessibility are correlated, being higher in GCSF conditions than in IL3, there is not a consistent causal connection between the two. *Cebpa* expression and the fold change of CRMs 7 and 18 peak early during the differentiation and change relatively little at later time points. The total accessibility of the enhancers changes little during early differentiation and peaks at later timepoints after PU.1 induction, which is consistent with PU.1’s role as a pioneer factor (Feng *et al.* 2008; Ghisletti *et al.* 2010; Barozzi *et al.* 2014; Minderjahn *et al.* 2020). The high resolution of the DNA accessibility profiles allowed us to examine the accessibility of individual nucleotides adjacent to TF binding sites, which is an indicator of TF occupancy (Baek *et al.* 2017; Bentsen *et al.* 2020). The accessibility of nucleotides neighboring specific C/EBP-family TF binding sites increases at early timepoints, showing that enhancer upregulation is driven by increased TF occupancy. These results support a model in which *Cebpa* is upregulated by GCSF treatment due to increased binding of sequence-specific TFs, and the later increase in total accessibility of the enhancers, while being a consequence of PU.1 binding, does not result in increased enhancer activity.

## Materials and methods

### PUER cell culture and differentiation protocol

PUER cells were cultured and differentiated as described previously (Dahl *et al.* 2003; Repele *et al.* 2019). Briefly, PUER cells were routinely maintained in complete Iscove’s Modified Dulbecco’s Glutamax medium (IMDM; Gibco, 12440061) supplemented with 10% FBS, 50 μM *β*-mercaptoethanol, 5 ng/mL IL3 (Peprotech, 213-13). PUER cells were differentiated into macrophages by adding 200 nM 4-hydroxy-tamoxifen (OHT; Sigma, H7904-5MG). Cells were differentiated into neutrophils by replacing IL3 with 10 ng/mL granulocyte colony stimulating factor (GCSF; Peprotech, 300-23) and inducing with 100 nM OHT after 48 h.

### Reporter knock-in cell line construction

#### Cas9 and donor vector design and cloning

Cas9 and the sgRNA were expressed from the pSpCas9(BB)-2A-GFP (PX458) plasmid from Addgene (Ran *et al.* 2013, #48138). The 20-nt guide (5^′^-ACTGGAGTTGCAGATCACGA-3^′^) targets a location (113053001–113053020 chr 6; mm10 coordinates) in the first intron of *Gt(ROSA)26Sor*. The guide was cloned into the vector by Gibson Assembly (Gibson Assembly Cloning Kit; New England BioLabs #E5510S) according to the manufacturer’s instructions. The vector was linearized by cutting at two BbsI sites that follow the hU6 promoter. The insert was made by amplifying part of the hU6 promoter with a reverse primer that includes the guide sequence, with an extra G at the 5^′^ end for enhanced expression, and sequence homologous to the pSpCas9(BB)-2A-GFP vector (Fwd primer: p-1; Rev primer: p-2; Supplementary Table S1).

The donor vectors were constructed in the pGL4.17[*luc2/Neo*] backbone from Promega (#E672A). DNA homologous to the genomic sequence flanking the Cas9 cleavage site at position 113053017 on chr 6 (“homology arms”) was cloned into the vector. Homology arm 1 (HA1; 113053018–113053656) was amplified from mouse genomic DNA with a forward primer that included the guide RNA target followed by GGG (Fwd primer: p-3; Rev primer: p-4; Supplementary Table S1). Both primers also contained homology to pGL4.17 for cloning into the SpeI site. HA2 (113052448–113053017) was amplified with a reverse primer that included the guide RNA target followed by GGG (Fwd primer: p-5; Rev primer: p-6; Supplementary Table S1). HA2 was cloned into the SalI site of the vector. Inclusion of two guide RNA target sites in the donor vector allowed its linearization in cells according to the double-cut donor strategy (Zhang *et al.* 2017). The *Cebpa promoter* was cloned between BglII and HindIII (Fwd primer: p-7; Rev primer: p-8; Supplementary Table S1). CRM 7 was cloned in the BamHI site (Fwd primer: p-9; Rev primer: p-10; Supplementary Table S1). CRM 18 was cloned in the BstBI site (Fwd primer: p-11; Rev primer: p-12; Supplementary Table S1). CRM 16 was cloned in the BstBI site (Fwd primer: p-13; Rev primer: p-14; Supplementary Table S1).

#### Antibiotic selection, limiting dilution, and screening

200,000 PUER cells were transfected with 500ng each of Cas9 and donor vector in SF buffer (Lonza, V4SC-2096) using program CM134 of the 4D-Nucleofector (Lonza). Cells were allowed to synthesize *Neo* protein product for 72 h before initiating antibiotic selection. Cells were selected with 1.6 mg/mL G-418 at a density of 2,500 cells/mL for 10 days. Limiting dilution was performed by seeding ∼30 cells per 96-well plate in G-418-free medium. Cells were fed on day 4 and colonies were transferred to a 24-well plate on day 8. On day 14, 200 μL of the cell suspension was washed twice in PBS, and genomic DNA was extracted with Quickextract solution (Lucigen, QE0905T) according to the manufacturer’s protocol.

Four primers used in three pairs were used to identify the knock-in lines in which the reporter gene had been inserted in a site-specific and biallelic manner. The “out” primers (out-1; out-2; Supplementary Table S1) flank the homology arms on chromosome 6, 213 bp after HA1 and 246 bp before HA2, respectively. The “in” primers are complementary to sequences in the insert (in-1; in-2; Supplementary Table S1). in-1 is complementary to the *Cebpa* promoter and elongates towards out-1, while in-2 is complementary to the *Neo* gene and elongates towards out-2. Amplicons of the predicted size resulting from PCR with in-1/out-1 and in-2/out-2 indicated successful HDR of each junction. PCR with the out-1/out-2 pair has three outcomes. If site-specific integration did not occur, the PCR is expected to produce a 1.7 kb amplicon. Biallelic site-specific integration is expected to result in amplicons ranging from ∼5.3 kb to ∼7.5 kb depending on composition of the insert. Monoallelic site-specific integration is expected to result in both the 1.7 kb and the longer amplicon. The in/out PCRs were carried out with Q5 polymerase (New England BioLabs, M0494S), while the out/out PCRs were carried out with PrimeSTAR GXL DNA Polymerase (Takara Bio, R051A). Seamless HDR was confirmed by sequencing the junctions between the insert and the mouse genome. This process generated 2, 5, 3, and 1 independent confirmed biallelic lines for Promoter, CRM 18, CRM 16, and CRM 7, respectively. Clones 9, 31, 7, and 7 were chosen for Promoter, CRM 18, CRM 16, and CRM 7, respectively for further experiments.

### Reporter assays

#### Bulk reporter assays

PUER cells were seeded at a density of $2.5\times 10^{5}$ cells/mL in 6 replicates before being differentiated as described above. Cells were sampled by washing twice in PBS and lysed in Glo Lysis Buffer (Promega, E2661) for 5 min at RT. The lysate was cleared by centrifugation at 12,000 rpm at 4^∘^C for 5 min. The Luciferase assay was performed with 100 μL of the cleared lysate (Steady Glo reagent, Promega, E2510) according to the manufacturer’s instructions. Total protein concentration was determined with the remaining 100 μL of the lysate using the BCA assay (Thermo Scientific, 23227) and a standard curve constructed with Albumin. Luminescence and absorbance were measured in a multimode plate reader (Beckman Coulter, DTX-880). Cell number was also estimated using the CellTox Green Cytotoxicity Assay (Promega, G8743) according to manufacturer’s instructions.

#### Single-cell reporter assays

Single-cell flow cytometry-based Luciferase measurements were done by intracellular immunostaining of Firefly Luciferase. 0.5–1 million cells of each reporter gene bearing line were sampled at each time point of the differentiation and spiked with ∼0.25 million cells of the Promoter line stained with CFSE (eBioscience, 65-0850). The CFSE-stained cells can be distinguished in the FITC channel during flow cytometry and their Luciferase signal provides an internal standard. In addition to reporter lines, PUER cells lacking Luciferase were also sampled to estimate background nonspecific staining during flow cytometry. The spiked samples were stained with the LIVE/DEAD Fixable Violet Dead Cell Stain Kit (Invitrogen, L34955) to exclude dead cells. Cells were fixed and permeabilized with BD Cytofix/Cytoperm Fixation and Permeabilization Solution (BD Biosciences, 554722) according to the manufacturer’s protocol. Cells were then stained for intracellular Firefly Luciferase with anti-Firefly Luciferase antibody (clone EPR17789, Abcam, ab185923) at a final concentration of 2.37 μg/mL (1:75 dilution) and polyclonal Goat Anti-Rabbit IgG H&L conjugated to Alexa Fluor 647 (Abcam, ab150083) at a final concentration of 1 μg/mL (1:2000 dilution). Fluorescence was recorded on a BD FACSymphony analyzer.

Luciferase expression, in units of undifferentiated Promoter expression, was calculated as follows. Let $f_{\mathrm{sample}}$ and $f_{\mathrm{sample}}^{s}$ be the median fluorescence of the stained Luciferase protein in the sample and the spiked Promoter internal standard, respectively. Also, let $f_{\mathrm{PUER}}$ and $f_{\mathrm{PUER}}^{s}$ be the median fluorescence in the PUER cells without Luciferase and Promoter internal standard they were spiked with respectively. The background nonspecific fluorescence in the sample was estimated as


$$b_{\mathrm{sample}}=f_{\mathrm{PUER}}\frac{f_{\mathrm{sample}}^{s}}{f_{\mathrm{PUER}}^{s}}.$$

The Luciferase expression in the sample was then computed as


$$L_{\mathrm{sample}}=\frac{f_{\mathrm{sample}}-b_{\mathrm{sample}}}{f_{\mathrm{sample}}^{s}-b_{\mathrm{sample}}}.$$

### Fast-ATAC

Fast-ATAC was conducted as described by Corces *et al.* (2016). 50,000 cells/sample were collected and centrifuged at 500 g for 5 min at 4^∘^C. The supernatant was removed and the resulting pellet was resuspended in a 50 μL transposition mixture consisting of 25 μL 2× TD buffer, 2.5 μL TDE1 (Illumina, FC-121-1030), 0.5 μL 1% digitonin (Promega, G9441), and 22 μL nuclease-free water. The resuspended cells were then incubated at 37^∘^C with shaking at 300 rpm for 30 min. DNA was purified using a Qiagen MinElute Reaction Cleanup Kit (Qiagen, 28204) and eluted in 10 μL of elution buffer (10 mM Tris-HCl, pH 8).

The transposed DNA library was amplified for a limited number of cycles as described by Buenrostro *et al.* (2013). First, 10 μL of transposed DNA was amplified in a 50 μL PCR reaction using NEBNext Ultra II Q5 Master Mix (New England BioLabs, M0544) for 5 cycles after a 5 min elongation step. Primers included adaptors for sequencing and were barcoded according to Illumina guidelines for pooling. The PCR reaction was held at 4^∘^C while 5 μL of its product was amplified in a 15 μL qPCR reaction with NEBNext Ultra II Q5 Master Mix and SYBR Green I (Invitrogen, S7563) at a final concentration of 0.5×. The number of PCR cycles needed to reach 1/4 of the maximum fluorescence was determined and the 50 μL PCR reaction was continued for as many additional cycles (9–11). The PCR product was purified using a Qiagen MinElute PCR Purification Kit and eluted in 20 μL elution buffer (10 mM Tris-HCl, pH 8). The libraries were then analyzed on an Agilent Bioanalyzer for QC before being pooled in equimolar amounts. The pooled libraries were sequenced in the 2×150 bp format to an average depth of 300 million reads/sample on a NovaSeq 6000 S4 (Illumina) by Psomagen Inc.

### Comparison of accessibility dynamics between endogenous and ROSA26 loci

ATAC-Seq libraries were prepared for the CRM 7 and CRM 18 reporter lines using Fast-ATAC as described above. We distinguished between the ROSA26 copy of the enhancer from the endogenous one and determined the accessibility of each by amplifying Tn5-generated fragments from the ATAC-Seq libraries using a combination of location-specific and Illumina primers and quantifying them with qPCR (Supplementary Fig. S13). The location-specific primers were complementary to the DNA sequence bordering the enhancers in each location, which corresponded to the pGL4.17[luc2/Neo] backbone sequence for the enhancer copy in ROSA26 (CRM 7: p-15, CRM 18: p-16; Supplementary Table S1) and chromosome 7 sequence for the endogenous copy (CRM 7: p-17, CRM 18: p-18; Supplementary Table S1). Tn5-generated fragments from the endogenous *Cebpa* promoter (p-19; Supplementary Table S1) were also quantified as an internal loading control for $\Delta C_{q}$ analysis as the promoter’s accessibility changes little during neutrophil differentiation (Fig. 3).

Location-specific accessibility was measured at three timepoints (−48, 0, and 96 h) during neutrophil differentiation in duplicate. For each sample, 3 qPCR reactions, for the endogenous enhancer copy, the ROSA26 enhancer copy, and the endogenous *Cebpa* promoter, were performed in (technical) triplicate. A 10 μL reaction was set up using 100 pg ATAC-Seq library DNA template, 417 nM location-specific primer (one of p15–p19; Supplementary Table S1), 417 nM Nextera index 1 primer complementary to the Tn5 adaptor (p-20; Supplementary Table S1), 1X Sybr Green I (Invitrogen, S7563), 1X NEBNext Ultra II Q5 Master Mix (New England BioLabs, M0544), and nuclease-free water. The libraries were then amplified for 40 cycles as described by Buenrostro *et al.* (2013).

After amplification, the difference between the ${\text{C}}_{q}$ of each enhancer copy and that of the internal loading control, the endogenous *Cebpa* promoter, $\Delta {\text{C}}_{q}={\text{C}}_{q}^{\mathrm{enhancer}}-{\text{C}}_{q}^{\mathrm{promoter}}$. Fold change was computed as $2^{-\Delta {\text{C}}_{q}}$. Technical replicates having a $\Delta {\text{C}}_{q}$ outside 1.5-times the interquartile range were removed prior to analysis.

### Data processing and analysis

Raw sequence files were tested for quality using FastQC (v. 0.11.5). Reads were trimmed to remove adaptor sequences and filtered to have a minimum phred score of 33 and a minimum length of 20 bp using Trimmomatic (v 0.39). Trimmomatic was run with the command-line options -PE -phred33 ILLUMINACLIP: <Trimmomatic-HOME>/adapters/NexteraPE-PE.fa:2:30:10:3:TRUE MINLEN:20, where <Trimmomatic-HOME> is the path to the Trimmomatic directory. The reads were aligned to the *mm10* genome, allowing for inserts up to 2 kb in length, using Hisat2 (v.2.2.0). Hisat2 was run with the command-line options -x -no-spliced-alignment -I 10 -X 2000. Duplicates were marked using the MarkDuplicates tool of Picard (v.2.23.1) package. Duplicate reads were removed during the read counting process in R.

#### Read counting

Tn5 cuts were counted in R (v. 4.1.0) using a custom script where each read was counted as one Tn5 cut. Properly paired and unique reads were read using the GenomicAlignments package (Lawrence *et al.* 2013) with filtering parameters isPaired = T, isProperPair = T, isUnmappedQuery = F, isSecondaryAlignment = F, isNotPassingQualityControls = F, isDuplicate = F, isSupplementaryAlignment = F. Tn5 leaves a 9 bp 5^′^ overhang (Supplementary Fig. S4a; Buenrostro *et al.* 2013; Li *et al.* 2019), so that the 5^′^ ends of a pair of reads resulting from the same transposition event map to different positions on the + and − strands. The read mapping to the + strand starts on the 1st nucleotide of the overhang whereas the one mapping to the − strand starts on the 9th nucleotide of the overhang. It is common to shift the Tn5 cut position by +4/ −5 bp for reads mapping to the +/− strand, respectively (Buenrostro *et al.* 2013). This however does not have the desired effect of assigning the same position to the two reads resulting from the same transposition event (Supplementary Fig. S4b). For both cuts to have the same location (5th nucleotide of the overhang), they must be shifted by +4/ −4 bp (Supplementary Fig. S4b,c). Finally, Tn5 cuts in each sample were normalized to their respective library size.

#### Bias correction

Tn5 transposase does not bind to and cut naked DNA uniformly but exhibits bias for certain motifs (Baek *et al.* 2017; Karabacak Calviello *et al.* 2019; Li *et al.* 2019; Bentsen *et al.* 2020). We tested two previously published Tn5 sequence bias correction methods, HINT-ATAC (Li *et al.* 2019) and BaGFoot (Baek *et al.* 2017), as well as a new method we developed in this study. Tn5 DNA sequence preferences were inferred from ATAC-Seq libraries prepared by transposing purified mouse genomic DNA with Tn5 [GSM2981009 (Senft *et al.* 2018), GSM1550786 (Pastor *et al.* 2014), GSM4048700 (Onimaru *et al.* 2021), and GSM2333650/GSM2333651 (Gray *et al.* 2017)].

##### Kmer-based bias inference

Tn5 binding preferences were inferred from purified DNA ATAC-Seq libraries by estimating the probability of Tn5 cutting at each possible hexamer. Since hexamers occur at different frequencies in the genome, the bias $b(w_{k})$ at hexamer $w_{k}$, where k=1,…,4096, was computed as the ratio of the frequency of observed Tn5 cuts $p_{c}(w_{k})$ to the frequency of the occurrence of the hexamer in the mouse genome $p(w_{k})$,


$$b(w_{k})=\frac{p_{c}(w_{k})}{p(w_{k})}.$$

All the purified DNA ATAC-Seq datasets were merged into one BAM file, and the MakeBiasCorrectionTableBAM function of the BagFoot package (Baek *et al.* 2017) was used with parameters np = 6, atac = T to compute the Tn5 bias at each hexamer.

##### HINT-ATAC bias correction

This was performed as described (Li *et al.* 2019). The number of bias-corrected cuts $x_{i}$ at position *i* were computed as


$$x_{i}=\frac{y_{i}+1}{{\hat{y}}_{i}\times \hat{b}(w(i))+1},$$

where $y_{i}$ are the observed cuts, w(i) is the hexamer at *i*, and b(w) is the Tn5 bias at hexamer *w*. ${\hat{y}}_{i}$ = $\frac{1}{50}\sum_{j=i-25}^{i+24}y_{j}$ is the mean of observed cuts in a 50 bp window around *i*. $\hat{b}(w(i))$ = $b(w(i))/\sum_{j=i-25}^{i+24}b(w(j))$ is the Tn5 bias at position *i* relative to the total bias over the 50 bp window (Supplementary Fig. S5, second panel).

##### BaGFoot bias correction

This was performed as described (Baek *et al.* 2017). The bias-corrected cuts $x_{i}$ at position *i* were computed as


$$x_{i}=\frac{y_{i}}{b(w(i))},$$

where $y_{i}$ are the observed cuts at position *i* and b(w(i)) is the bias of the hexamer w(i) at position *i* (Supplementary Fig. S5, third panel).

##### Our bias correction method

HINT-ATAC corrects the observed cuts with a factor that depends on the bias at the position relative to average bias in a 50 bp window and the average number of cuts in the window. As a result, it does not correct fine-scale bias at the resolution of individual binding sites and also adds one cut to each position even if it had no cuts originally (Supplementary Fig. S5). While BaGFoot does not average the cuts or the bias, it can amplify noise at low-accessibility nucleotides which could mask protected regions.

We developed a method that corrects bias at the resolution of individual nucleotides without amplifying noise at positions with low counts. The method adjusts the cuts at individual nucleotides but only for positions that have more cuts than the background level. We modeled the cuts at each position with the Poisson distribution $P(y;\lambda )=\frac{\lambda^{y}}{y!}e^{-\lambda }$, where *λ* is the mean number of cuts in the sample across the genome. We correct the bias for a position *i* if $P(y_{i};\lambda )<\frac{0.01}{G}$, where *G* is the size of the mouse genome (Bonferroni correction) so that the corrected cuts are


$$x_{i}=\frac{y_{i}}{b(w(i))},$$

where b(w(i)) is the bias of the hexamer at *i* (Supplementary Fig. S5, fourth panel).

### Identification of new binding sites in *Cebpa* enhancers

Protected regions were identified as runs of 5–50 bp consecutive low-accessibility nucleotides bordered by positions having more cuts than an enhancer-specific threshold. Cuts were pooled over all samples for this analysis. The thresholds were 400, 100, 40, or 200 in the promoter, CRM 7, 16, or 18, respectively. Putative TFs binding to the protected regions were identified using TRANSFAC’s *Match* tool (Kel *et al.* 2003, https://gene-regulation.com) using immune-specific position weight matrices (PWMs).

## Results

### Enhancer activity

We utilized CRISPR/Cas9 homology directed repair (HDR) with a “double-cut” donor strategy (Zhang *et al.* 2017) to create transgenic PUER cell lines bearing synthetic reporter loci in the ROSA26 locus (Fig. 1d). We created a line with the *Cebpa* promoter driving *luc2* reporter gene transcription but lacking an enhancer (promoter line), and lines bearing an enhancer, CRM 7, 16, or 18, in addition to the promoter (referred to as CRM 7, 16, and 18 lines, respectively). Our experimental strategy (see *Materials and Methods*) ensured that the synthetic loci were integrated into the same location in a biallelic manner and that the integration was seamless. Multiple biallelic clones derived from independent HDR events had reporter expression within 30% of each other (Supplementary Fig. S1a) and the expression of the enhancer-bearing lines differed significantly from the promoter line ($P<5\times 10^{-5}$; Wilcoxon Rank-Sum test; Supplementary Fig. S1b). A representative clone was selected for further time series experiments (see *Materials and Methods*).

We measured reporter expression during the differentiation of PUER cells by Luciferase assay at several time points. PUER cells are maintained in IL3 medium and can be differentiated into macrophage-like cells over a period of 7 days by activating the PU.1-estrogen receptor fusion protein with 4-hydroxy-tamoxifen (OHT) (Dahl *et al.* 2003; Bertolino *et al.* 2016; Repele *et al.* 2019). Replacing IL3 medium with GCSF medium for 48 h prior to OHT induction causes PUER cells to differentiate into neutrophils over a 7-day period. We regard OHT induction as the initiation of differentiation so that undifferentiated PUER cells correspond to the 0 h time point in IL3 conditions and the −48 h time point in GCSF conditions (Fig. 1b).

The mRNA and protein expression of the endogenous *Cebpa* gene has a characteristic temporal profile, being upregulated about 2-fold relative to undifferentiated cells during the 48-h pretreatment with GCSF, peaking at 24 h after OHT induction and then declining subsequently (Fig. 1c; Dahl *et al.* 2003; Repele *et al.* 2019). In contrast, *Cebpa* expression is relatively constant in IL3 conditions so that the GCSF response accounts for the neutrophil-specific upregulation of the gene.

During neutrophil differentiation, reporter gene expression per cell (Fig. 1e), estimated by normalizing the observed Luciferase luminescence to total protein, shows a transient upregulation peaking during the first 12 h of GCSF pretreatment for all the reporter genes. CRMs 18 and 7 have the largest effect, peaking 50% and 80% over their levels in undifferentiated cells respectively, while the promoter peaks at a 40% increase over its level in undifferentiated cells. With the exception of the −40 h timepoint, the changes in expression at all other timepoints are much larger than the variation between independent lines (p<0.01; Wilcoxon Rank-Sum test; Supplementary Fig. S2). The activity of the CRMs may be characterized by computing the fold change in expression relative to the promoter. As expected, all three enhancers drove reporter expression at levels higher than the promoter in undifferentiated PUER cells (Fig. 1f; Repele *et al.* 2019), with CRMs 7 and 18 having a fold change of ∼2. The fold change of CRMs 7 and 18 increases to ∼3.5 during the GCSF pretreatment and declines upon OHT induction, staying above 1 at all times. In contrast, the fold change of CRM 16 declines to less than 1 during neutrophil differentiation, suggesting a dominant negative effect on the transcription of the promoter. We checked these measurements by an independent method we developed to assay reporter gene expression at the single-cell level using flow cytometry (see *Materials and Methods*). The single-cell reporter data have unimodal distributions of Luciferase expression (Supplementary Fig. S3b) showing that gene expression is not heterogeneous. The time series of median expression (Supplementary Fig. S3c) agree with the bulk measurements. Overall, the temporal pattern of the fold change of CRMs 7 and 18 (Supplementary Fig. S3d) matches that of endogenous *Cebpa* during GCSF pretreatment, showing that these two CRMs are primarily responsible for the gene’s neutrophil-specific upregulation.

OHT induction in IL3 conditions causes a comparable upregulation of all the reporter genes, so that the fold change remains relatively constant for CRMs 16 and 18 and declines from ∼2 to ∼1 for CRM 7 (Fig. 1f). As in GCSF conditions, the temporal pattern of fold change matches the relatively constant expression of the endogenous gene in IL3 conditions.

### Accessibility landscape of *Cebpa*

In order to discover the regulatory mechanisms driving the temporal patterns of reporter expression, we profiled the accessibility of the *Cebpa* locus with deep sequencing at several time points during the differentiation of PUER cells into macrophages and neutrophils. The accessibility of regulatory regions is regarded as a determinant of gene expression since it could influence the occupancy of TF binding sites (Klemm *et al.* 2019; Coux *et al.* 2020). Conversely, TFs influence the accessibility of enhancers by competing with and displacing nucleosomes (Adams and Workman 1995; Anderson *et al.* 2002; Mirny 2010). Profiling accessibility with deep sequencing has the potential to reveal TF binding sites as footprints (Buenrostro *et al.* 2013; Gusmao *et al.* 2014; Yardımcı *et al.* 2014; Gusmao *et al.* 2016; Karabacak Calviello *et al.* 2019; Bentsen *et al.* 2020) and the *cis*-regulatory logic of *Cebpa* regulatory regions.

We sampled cells in duplicate at multiple stages of PUER differentiation (Fig. 1b) and profiled their DNA accessibility using a variation of the classical ATAC-seq method (Buenrostro *et al.* 2013) known as Fast-ATAC (Corces *et al.* 2016). Following sampling, DNA libraries were prepared following the Fast-ATAC protocol (see *Materials and Methods*) and were sequenced to approximately 300 million reads/sample with paired-end sequencing. The sequences were then filtered, trimmed, and aligned to the *mm10* genome. We counted each read as one Tn5 cut. Tn5 leaves a 9 bp 5^′^ overhang (Supplementary Fig. S4; Sato *et al.* 2019), so that the 5^′^ ends of reads resulting from the same transposition event are mapped to different locations. We shifted the position of the cut to the center of the overhang by adding +4/−4 bp if the read mapped to +/− strand, respectively (Supplementary Fig. S4b,c). Tn5 transposition is not uniformly random on naked DNA and these biases can obscure TF footprints. Methods for correcting bias either average over 50–100 bp (Li *et al.* 2019) and thus do not correct bias at single-nucleotide resolution or do not average and amplify noise at low accessibility sites (Baek *et al.* 2017). We developed a new method that overcomes these limitations (see *Materials and Methods* and Supplementary Fig. S5) in estimating bias from purified mouse genomic DNA ATAC-Seq libraries (Pastor *et al.* 2014; Gray *et al.* 2017; Senft *et al.* 2018; Onimaru *et al.* 2021) and correcting it. Finally, corrected Tn5 cut counts were normalized to each sample’s library size.

**Fig. 2.**
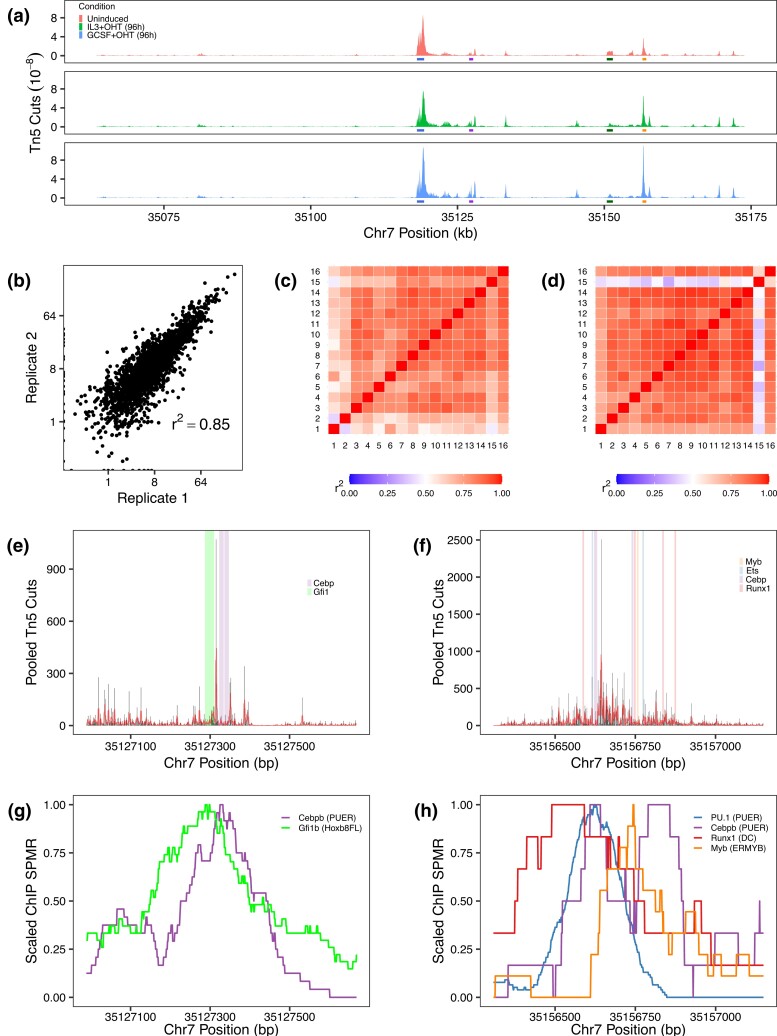
Locus- and enhancer-level accessibility profiles. a) The chromatin accessibility profile of a 110 kb region (locus) centered on the *Cebpa* TSS in uninduced PUER cells, 96 h after OHT induction in IL3 (macrophage), and 96 h after OHT induction in GCSF (neutrophil). b) Tn5 cuts at each nucleotide in the 110 kb locus averaged with a 100 bp sliding window. The two IL3+OHT (96 h) replicates are compared. c) Pearson correlation coefficients of Tn5 counts in CRM 7, averaged with a 10bp sliding window, computed between each pair of samples. d) Pearson correlation coefficients of Tn5 counts in CRM 18, averaged with a 10 bp sliding window, computed between each pair of samples. e) Pooled single-nucleotide accessibility profile of CRM 7. Bars are the sum of Tn5 counts over all the samples. The line is a 3 bp sliding-window average. Shaded regions show empirically verified known binding sites. f) Pooled single-nucleotide accessibility profile of CRM 18. g) Scaled ChIP-Seq signal of C/EBP*β* in PUER cells (GSM538010) and Gfi1b in Hoxb8FL cells (GSM2231904) in CRM 7. h) Scaled ChIP-seq signal of C/EBP*β* (GSM538010) and PU.1 (GSM538004) in PUER cells, Runx1 in dendritic cells (GSM881115), and Myb in ERMYB cells (GSM549341) in CRM 18.

The accessibility profile of a 110 kb region centered on the *Cebpa* TSS (*Cebpa locus*) is highly correlated between replicates (Fig. 2b) and time points (Fig. 2a). Neither newly accessible regions appear nor are any accessible regions lost during differentiation. Instead, we observed relatively smooth quantitative changes in the accessibility of different regions in the locus (Fig. 2a), which is consistent with similar analyses in other cell types (Kim *et al.* 2021). In undifferentiated PUER cells, the *Cebpa* promoter was the most accessible region, followed in descending order by CRM 18, 7, and 16. Interestingly, while *Cebpa* expression in GCSF conditions is roughly 2-fold its value in IL3 (Fig. 1c), the accessibility of the *Cebpa* promoter is comparable between the conditions. The accessibility of two enhancers, CRMs 7 and 18, both increased approximately 2-fold during differentiation in neutrophil conditions suggesting correlation between the upregulation of *Cebpa* expression and the chromatin state of the enhancers.

**Fig. 3.**
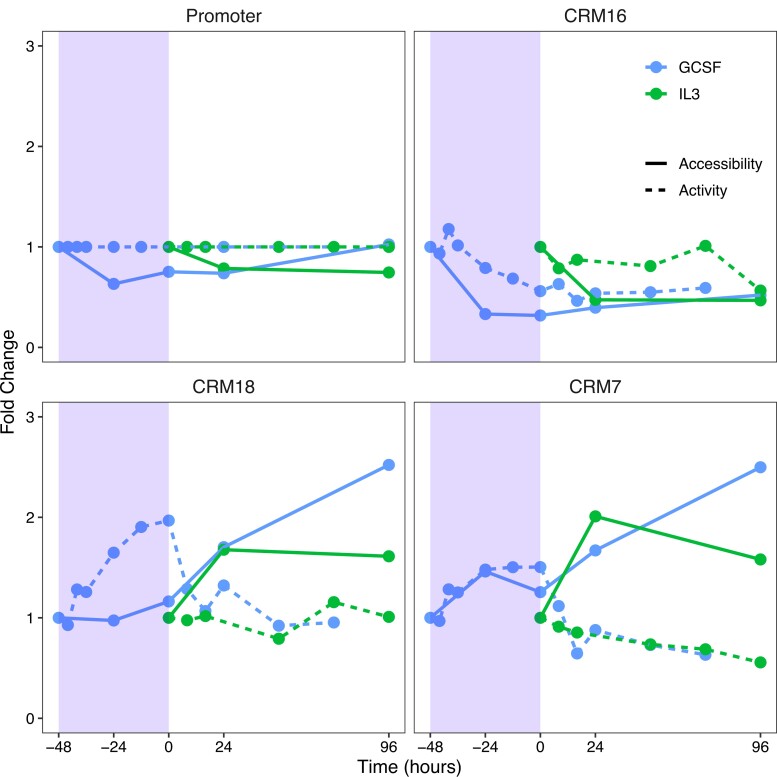
The dynamics of enhancer accessibility and expression. Fold change in the expression of enhancer-bearing reporters relative to promoter (dotted) and total accessibility (solid) are plotted together. Fold change and accessibility were normalized to their values in undifferentiated PUER cells. Differentiation in macrophage (IL3) and neutrophil (GCSF) conditions is shown. The shaded region shows the period of GCSF pretreatment prior to induction with OHT during neutrophil differentiation.

### *Cebpa* enhancer structure

To further understand the regulatory logic of *Cebpa*’s enhancers, we analyzed their accessibility profiles at high resolution. The accessibility profiles of the promoter, CRM 7, and CRM 18 were highly correlated between samples (Fig. 2c,d and Supplementary Fig. S6a), similar to the 110 kb region. CRM 16 is an exception, having low correlation between samples (Supplementary Fig. S6b), which could be the result of the dynamic changes in profile during differentiation (Supplementary Fig. S7) or noise due to relatively lower accessibility (see below). The high correlation between samples suggests that the chromatin structure of the promoter and CRMs 7 and 18 remains relatively constant and that the enhancers are bound at the same sites throughout differentiation. The high correlation of CRMs 7 and 18 and the promoter allowed us to pool the samples by summing the Tn5 cuts at each nucleotide, resulting in a very high depth of coverage in the accessible regions of the genome. For example, after pooling, CRM 18 has a total of 39,691 reads over its 642 bp length, resulting in a coverage of ∼62 reads/nucleotide. The pooled accessibility profile of the enhancers (Fig. 2e,f and Supplementary Fig. S6c,d) consists of many short high-accessibility “islands” 1–5 bp in length interspersed between 5–50 bp-long regions having nearly zero accessibility. Such high–low–high accessibility patterns are regarded as TF footprints (Buenrostro *et al.* 2013; Li *et al.* 2019), although there has been mixed success in detecting them at an individual level (Baek *et al.* 2017; Li *et al.* 2019; Bentsen *et al.* 2020).

To determine whether these high–low–high patterns are, in fact, TF binding sites, we mapped previously characterized high-confidence binding sites to each enhancer. These sites have been identified and validated previously with multiple methods such as DNase I footprinting, EMSA, ChIP, and site-directed mutagenesis coupled to reporter assays (Legraverend *et al.* 1993; Guo *et al.* 2012; Cooper *et al.* 2015; Bertolino *et al.* 2016; Repele *et al.* 2019). All previously identified binding sites overlapped with the footprints (Fig. 2e,f, Supplementary Figs. S6c,d and S21). Nucleotides in previously validated binding sites had significantly fewer cuts than those lying outside binding sites (Supplementary Fig. S8; Wilcoxon Rank-Sum test; Promoter: $p=10^{-61}$, CRM 18: $P=1.7\times 10^{-4}$, CRM 7: $P=7.7\times 10^{-30}$). CRM 7 has protected binding sites for both Gfi1 and C/EBP-family TFs while CRM 18 has protection by a multitude of TFs including Myb, Ets-family, C/EBP-family, and Runx1, showing that these sites are occupied in PUER cells. We further validated the footprints against publicly available ChIP-Seq datasets, where available, for the TFs predicted to bind them, PU.1 and C/EBP*β* (PUER; Heinz *et al.* 2010), Runx1 (dendritic cells; Garber *et al.* 2012), Myb (ERMYB; Zhao *et al.* 2011), and Gfi1b (Hoxb8FL; Nestorowa *et al.* 2016) and found that the maxima of all of the ChIP peaks were located at or near the footprints (Fig. 2g,h and Supplementary Fig. S6e,f). The strong agreement between the Tn5-protected regions and independent evidence of TF binding sites shows that the high depth-of-coverage ATAC-Seq data can be used to track TF binding at the resolution of individual sites. While all the previously known binding sites overlap footprints, the regulatory elements contain additional footprints that do not overlap known binding sites, suggesting a much more complex regulatory scheme than described previously (Guo *et al.* 2012, 2014; Cooper *et al.* 2015; Bertolino *et al.* 2016; Repele *et al.* 2019).

We determined which TFs might bind to the novel protected regions by scoring their sequences with position weight matrices (PWMs). The protected regions were identified as 5–50 bp consecutive nucleotides having less than a threshold number of cuts (see Materials and Methods and Supplementary Figs. S9–S12). With this criterion, CRMs 7 and 18 had 15 and 21 potential footprints, respectively, including previously known binding sites. The sequence of each novel footprint was scored with a set of immune-cell specific TF PWMs from TRANSFAC (Matys *et al.* 2006). 5 footprints matched C/EBP-family, Runx1, Ikzf1, and Jun/Fos on CRM 7 (Supplementary Fig. S9) while 7 footprints matched Runx1, Ikzf1, MZF1, Jun/Fos, GATA-3, and Sp1 on CRM 18 (Supplementary Fig. S10). There is a preponderance of matches to Runx1 and C/EBP-family TF sites (Supplementary Table S2). This type of architecture, where an enhancer has many low-affinity binding sites for a single TF, has been shown to increase enhancer sensitivity through a process known as suboptimization (Farley *et al.* 2015; Kim *et al.* 2021). The large number of sites for the two TFs predicts that CRMs 7 and 18 are highly sensitive to the expression of Runx1 and C/EBP-family TFs.

### Accessibility dynamics and the *cis*-regulatory logic of *Cebpa* enhancers

Having determined the architecture of *Cebpa* regulatory regions at binding site resolution, we next compared the dynamics of accessibility and reporter expression. We computed total accessibility as the sum of Tn5 cuts over each regulatory element during differentiation (Fig. 3). Since enhancers boost gene expression to a level higher than that driven by the promoter itself, we compared their accessibility to the fold change of the enhancer-bearing reporters (Fig. 1f). While *Cebpa* is upregulated 2-fold in GCSF conditions (Fig. 1c), the total accessibility of its promoter does not change much, declining slightly during pretreatment and then recovering after OHT induction. We observed a correlation between enhancer accessibility and fold change for all the enhancers. CRMs 7 and 18 have both higher fold change and higher accessibility in GCSF compared to IL3, whereas both fold change and accessibility of CRM 16 decrease in time (Fig. 3).

Despite the correlation, a strict causal link between total accessibility and fold change is lacking. The fold change of CRMs 7 and 18 increases and peaks at earlier time points during GCSF pretreatment but total accessibility either does not change at all (CRM 18) or increases slightly (CRM 7). Similarly, while the total accessibility of CRMs 7 and 18 peaks 96 h after OHT treatment in GCSF conditions, their fold change declines over that interval. Therefore, even though the total accessibility of the two enhancers at 96 h is ∼2.5 times its value at −48 h, the upregulation relative to the expression of the promoter at 96 h is comparable to or lower than its initial value.

It is possible that the accessibility dynamics of the enhancers are altered by their placement in the ROSA26 locus so that accessibility increases during the GCSF pretreatment period and then declines at 96 h. We checked this possibility by profiling the accessibility dynamics of CRMs 7 and 18 in the ROSA26 locus and comparing with the endogenous enhancers. We distinguished between the ROSA26 copy of the enhancer from the endogenous one by amplifying fragments using a combination of location-specific and Illumina primers and quantifying them with qPCR (Supplementary Fig. S13; see *Materials and Methods*). First, we checked the validity of the approach by comparing the accessibility dynamics of the endogenous enhancers between location-specific ATAC-qPCR and ATAC-Seq. The accessibility of both enhancers changes little during GCSF pretreatment but is ∼2.5-($P=8.7\times 10^{-3}$; one-tailed Wilcoxon ranksum test) and ∼1.75-fold higher ($P=8.7\times 10^{-3}$) for CRM 18 and 7, respectively (Supplementary Fig. S14a,c), recapitulating the dynamics previously observed with ATAC-Seq (Fig. 3).

The enhancer copies in the ROSA26 locus exhibit the same pattern of accessibility dynamics as the endogenous ones (Supplementary Fig. S14a,c). CRM 18’s accessibility changes little during GCSF pretreatment when the fold change driven by the enhancer is highest (Fig. 3), while accessibility peaks at 96 h ($P=8.7\times 10^{-3}$ and P=0.066 compared to 0 and −48 h timepoints respectively). The accessibility of CRM 7 is also the highest at 96 h although the difference is not statistically significant (P=0.21). Another way to check whether accessibility dynamics of the ROSA26 copy are the same as the endogenous ones is to profile the ratio of accessibility between the two locations (Supplementary Fig. S14b). The difference in the quantification cycle between the ROSA26 and endogenous copies of either enhancers ($\Delta {\text{C}}_{q}$) does not change in time, and remains within 15% of the initial value at 96 h, supporting the conclusion that accessibility dynamics do not differ between the two genomic locations. These results show that the accessibility of the enhancers in the reporter gene does not change much during GCSF pretreatment, when the fold change of gene expression is the highest, while accessibility peaks at 96 h, when the fold change has declined ∼2-fold from its peak at 0 h to its value in undifferentiated cells.

**Fig. 4.**
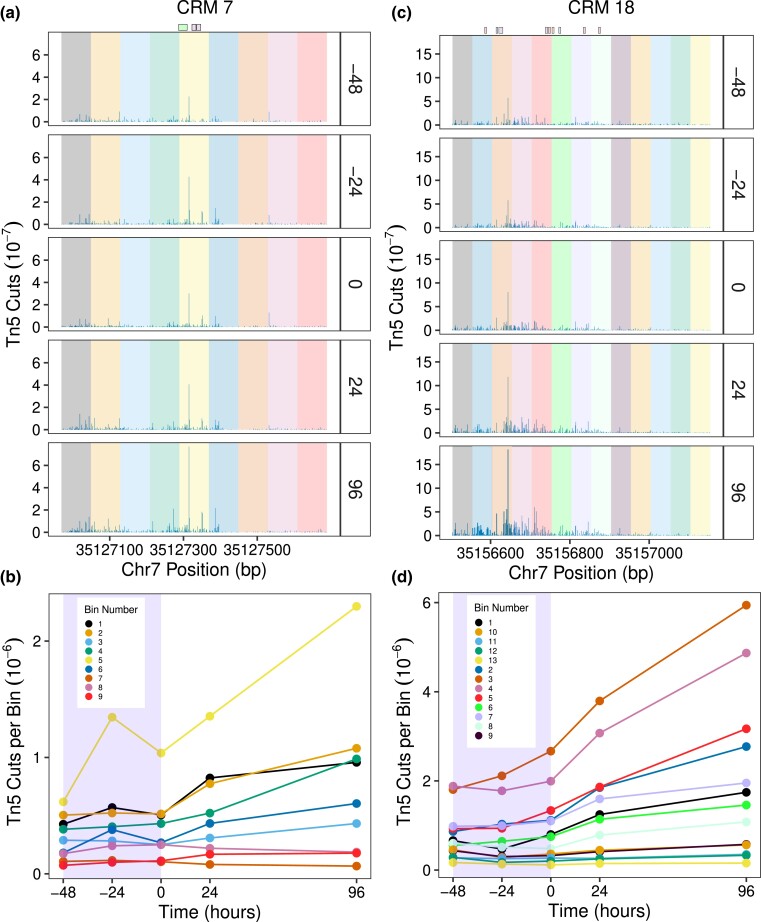
Binding site-resolution dynamics of enhancer accessibility during differentiation. a, c) The panels show Tn5 cuts at each nucleotide of CRMs 7 (a) and 18 (c) at different time points in GCSF conditions. Each enhancer has been uniformly divided into bins shown as shaded regions. TF binding sites are shown at the top; see Fig. 2e,f for legend. Replicates have been pooled and normalized by library size. b, d) Time series of average accessibility in each bin for CRMs 7 (b) and 18 (d). The shaded region shows period of GCSF pretreatment prior to induction with OHT during neutrophil differentiation.

The lack of a causal link between total accessibility and fold change led us to examine the accessibility dynamics of individual nucleotides within CRMs 7 and 18. The accessibility of a few positions does, in fact, increase during GCSF pretreatment (Fig. 4a,c, Supplementary Figs. S15 and S16). We divided the two enhancers uniformly into bins and characterized the accessibility within each bin (Fig. 4b,d). Of 9 bins in CRM 7, the accessibility of only one, bin 5, increases during the pretreatment period, while all but three respond after OHT treatment. Notably, bin 5 contains binding sites for C/EBP-family TFs. Of 13 bins in CRM 18, bins 3 and 5, also containing C/EBP-family TF sites, increase in accessibility during GCSF pretreatment, while 9 of 13 are OHT responsive. The change in average accessibility between consecutive timepoints is 6–10 fold higher in the vicinity of C/EBP sites as compared to the remainder of the enhancer (Supplementary Fig. S17a,b). These data indicate that only the accessibility of nucleotides adjacent to C/EBP binding sites increases during GCSF treatment while OHT treatment results in a broad increase in accessibility across each enhancer.

The accessibility of binding site-adjacent nucleotides to Tn5 increases with TF occupancy (Supplementary Fig. S18; Baek *et al.* 2017; Bentsen *et al.* 2020), implying that the observed increase in accessibility is a result of increased occupancy of C/EBP binding sites. The early increase in accessibility, being localized to a few nucleotides, is a small proportion of the total and therefore does not increase it appreciably. OHT induction, in contrast, results in a broad increase in accessibility across the length of the enhancers (Fig. 4, Supplementary Figs. S7, S15, S16, and S19). The main consequence of OHT induction is the activation of the PUER protein, resulting in an increase in PU.1 binding at its sites (Supplementary Figs. S18, S20 and Fig. 2f,h). The broad increase in accessibility is most likely a result of PU.1’s non-conventional pioneering activity as it is known to be capable of displacing nucleosomes to increase the accessibility of enhancers (Barozzi *et al.* 2014; Minderjahn *et al.* 2020). These data show that the OHT-dependent increase in the accessibility of the enhancers does not translate into an appreciable increase in the upregulation driven by them (Fig. 3).

## Discussion

The transcription of developmental genes is regulated in a complex scheme, involving multiple enhancers (Hong *et al.* 2008; Marinić *et al.* 2013; Whyte *et al.* 2013; Choi *et al.* 2021), and multiple TFs/binding sites per enhancer (Wilson *et al.* 2010; Kim *et al.* 2013; Kittler *et al.* 2013; Cooper *et al.* 2015). This makes it challenging to determine which particular TF or subset of TFs causally govern gene expression in particular tissues or at particular time points during development. We utilized coupled time series datasets of DNA accessibility and gene expression to find causal links between the chromatin state and gene expression of *Cebpa* enhancers.

The comparisons of total accessibility and gene expression did not support a causal link between the two. We computed the fold change in the expression of the reporter bearing both an enhancer and the *Cebpa* promoter to that of a promoter-only reporter as a measure of the activity of the enhancer. Accessibility and fold change do not follow a consistent pattern when comparing enhancers. While CRM 18 has roughly 2-fold higher accessibility than CRM 7 in undifferentiated cells (Supplementary Figs. S15 and S16), its fold change is slightly lower than CRM 7’s (Fig. 1f). There is a lack of correspondence when comparing accessibility and fold change in time as well. Although there is a general correlation between the total accessibility of CRM 7 and 18 and the fold change in reporter expression driven by them, where both properties increase during differentiation, the sequence of events shows that increased accessibility could not be causing greater fold change. The increase in accessibility follows the increase in fold change instead of preceding it (Fig. 3) at both the ROSA26 and endogenous loci (Supplementary Fig. S14a,c). The discrepancy is particularly striking in the period following PU.1 activation by OHT, which accounts for the bulk of the increase in accessibility both at the endogenous and ROSA26 loci (Fig. 3 and Supplementary Fig. S14c). Both the CRMs have PU.1 binding sites in their vicinity (Fig. 2f,h and Supplementary Fig. S20) and the increased accessibility is consistent with PU.1’s role as a pioneer TF (Feng *et al.* 2008; Ghisletti *et al.* 2010; Barozzi *et al.* 2014; Minderjahn *et al.* 2020). The increase in accessibility of CRM 7 was lower in the ROSA26 locus compared to the endogenous one and the difference was not statistically significant (Supplementary Fig. S14a,c). This agrees with the fact that the PU.1 binding site near CRM 7 in the endogenous locus was not included in the ROSA26 reporter (Fig. 2e and Supplementary Fig. S20). Despite the small differences in accessibility between the three time points, the fold change of CRM 7 first increases 2-fold during GCSF pretreatment and then declines equally precipitously after OHT induction, showing a lack of correspondence between accessibility and gene expression.

This result is surprising since DNA accessibility is thought to regulate transcription by limiting the access of TFs to their binding sites (Kornberg and Lorch 1992; Bartsch *et al.* 1996; Mellor 2005; Boyle *et al.* 2008; Thurman *et al.* 2012; Buenrostro *et al.* 2013). A potential reason for the lack of a causal link between total accessibility and fold change is that accessibility is a limiting factor only when it is very low and nucleosome occupancy is high. Once accessibility increases beyond a threshold value, other factors such as TF occupancy and activity, might drive gene expression. In support of this explanation, we observed a coordinate reduction in both the accessibility and fold change of CRM 16, which has a relatively low fold change of 1.17–1.38 during the initial 12 h of GCSF pretreatment (Fig. 1f) and could be near the hypothesized accessibility threshold. In contrast, CRMs 7 and 18 have a fold change of ∼2 in undifferentiated PUER cells and are perhaps above the accessibility threshold. For these enhancers, TF occupancy, rather than the overall accessibility of the enhancer, drive the increase in fold change during GCSF pretreatment (Fig. 4 and Supplementary Fig. S17). To summarize, the apparent disconnect between total accessibility and fold change might be reconciled with a model in which, after an initial opening, the upregulation caused by enhancers is primarily driven by TF occupancy and activity.

Although TF binding sites can be detected in DNase-Seq and ATAC-Seq data (Feng *et al.* 2008; Gusmao *et al.* 2014, 2016), there has been mixed success in identifying individual or even aggregate footprints for some TFs (Baek *et al.* 2017; D’Oliveira Albanus *et al.* 2021). Using the accessibility profiles based on pooled data (Fig. 2e,f and Supplementary Fig. S6c,d) we were able to recover all of the previously known binding sites as individual footprints. Whether this can be generalized to genes other than *Cebpa* will be the subject of future work. However, success in correctly identifying 21 binding sites for about 10 different TFs provides hope that high depth-of-sequencing ATAC-Seq libraries coupled with the bias correction method developed here might improve the genome-wide detection of binding sites. While the *Cebpa* promoter and enhancers were already known to have a complex organization (Cooper *et al.* 2015; Bertolino *et al.* 2016; Peng *et al.* 2019; Repele *et al.* 2019), these accessibility profiles reveal many new footprints, corresponding to about 6 TFBSs per element. In particular, CRM 7 is predicted to bind C/EBP at 3 additional sites (Supplementary Fig. S9), and CRM 18 is predicted to bind Runx1 at 5 new sites (Supplementary Fig. S10). This composition of the enhancers, with multiple weak sites instead of a few strong sites is reminiscent of “suboptimization” (Farley *et al.* 2015; Kim *et al.* 2021), which allows specific and sensitive control of gene expression in response to the TF.

We found that the temporal pattern of accessibility was largely conserved between the endogenous and ROSA26 copies of the enhancers. This result agrees with previous observations that when promoters are placed in different genomic environments, they maintain their relative strength relationships despite great variation in absolute expression (Hong and Cohen 2022). This implies that, even if there are any genomic location-specific effects, they are limited to absolute expression and activity and do not affect the temporal dynamics. A limitation of our experimental design is that the enhancers were not placed at the same distance from the promoter as in the endogenous *Cebpa* locus. A recent study utilized the piggyBac transposon to vary the distance between an enhancer and its cognate promoter showed that enhancer activity decreased with distance, with 60%–80% activity at ±40 kb (Zuin *et al.* 2022). The fold change measurements therefore likely overestimate enhancer strength by virtue of being placed closer than in the endogenous locus, although this effect should be uniform across time points.

Developmental genes have complex regulatory schemes with multiple enhancers, each potentially bound by several TFs at a large number of sites. Although the regulatory logic of a few well-studied loci is well understood, the complexity of regulatory architecture has limited our ability to infer cause-effect relationships. Our results show that profiling chromatin state at high genomic and temporal resolution coupled to reporter data can provide insight into the causality of regulatory events directing differentiation.

## Supplementary Material

jkad269_Supplementary_Data

## Data Availability

ATAC-Seq data are available at GEO with the accession number: GSE227645. Plasmids are available upon request. Supplementary material are available at *G3* online.
