# Supplementary material for: The contributions of DNA accessibility and transcription factor occupancy to enhancer activity during cellular differentiation
Source: G3 (Bethesda). 2023 Nov 22;14(2):jkad269. doi: 10.1093/g3journal/jkad269 (PMC11090500; doi:10.1093/g3journal/jkad269)
Supplement: jkad269_Supplementary_Data [file jkad269_supplementary_data.zip › Supplemental_Material_G3-2023-404635.pdf]

The contributions of DNA accessibility and transcription factor occupancy to enhancer activity during cellular differentiation

Supplementary Information

Trevor Long†, Tapas Bhattacharyya†, Andrea Repele, Madison Naylor, Sunil Nooti, Shawn Krueger, & Manu*

Department of Biology,
University of North Dakota,
10 Cornell Street, Stop 9019, Grand Forks, ND 58202-9019, U.S.A.

†Equal contribution
*Corresponding author

E-mail: manu.manu@und.edu

S1 Supplementary Information Captions

Table S1. Primer sequences used in vector construction and screening.

Table S2. Chromosomal locations of preliminary novel transcription factor binding sites.

Fig S1. Comparison of Luciferase activity in independent lines generated by CRISPR/Cas9 HDR. A. Luciferase activity for undifferentiated cells in all confirmed biallelic lines. The luminescence was normalized to cell number estimated using the CellTox assay. In the boxplots, the horizontal line indicates median over 8 replicate measurements, the bottom and top edges are the first and third quartiles, while the vertical lines indicate 1.5 times the interquartile range. Individual replicate measurements are shown as points. **B.** Boxplots include data for all lines for a particular reporter. p-values for the one-sided Wilcoxon Rank-Sum test between each enhancer and the promoter, with the alternative that the promoter has greater expression, are shown above brackets.

Fig. S2. The changes in reporter expression during differentiation are significantly larger than the variation between independent lines. The distribution of reporter expression is plotted as boxplots at each timepoint of the differentiation. Boxplots are defined in Figure S1A. The data for undifferentiated cells (-48 hrs) include measurements from all independent lines created for a particular enhancer (Fig. S1A). p-values are indicated by asterisks; *: $10^{-2} \leq p < 0.05$, **: $10^{-3} \leq p < 10^{-2}$, ***: $10^{-4} \leq p < 10^{-3}$, ****: $10^{-5} \leq p < 10^{-4}$. **A–C.** GCSF conditions. **D–F.** IL3 conditions. **A,D** CRM 18. **B,E.** CRM 7. **C,F.** CRM 16.

Fig. S3. Reporter expression and fold change measured by flow cytometry.A. Dot-plot of CFSE and Alexa Fluor 647 (Luciferase secondary) fluorescence showing the CFSE^{+} spiked internal control (undifferentiated Promoter) and the CFSE^{-} sample (CRM 7 in this example). Cells have been gated to exclude debris, doublets/clusters, and dead cells. **B.** Histograms of Luciferase fluorescence intensity of undifferentiated cells of the PUER unedited founder line (Luc^{-}), Promoter, CRM 18, CRM 16, and CRM 7. **C.** Median Luciferase expression in units of CFSE^{-} spiked undifferentiated promoter during differentiation in GCSF conditions. CRM 18 and Promoter were sampled at high frequency during the first 24 hrs of GCSF pre-treatment and show strong upregulation consistent with bulk Luciferase data (Fig. 1E). **D.** Fold change in median expression relative to the expression of the Promoter, showing upregulation of CRMs 7 and 18 during GCSF pre-treatment.

Fig S4. Comparison of different strategies for counting Tn5 cuts. A. Schematic of cleavage by the Tn5 transposase. Adaptor 1 and 2 are Illumina read 1 and 2 adaptors respectively. The pair of green strips represents double stranded genomic DNA. Black triangles are sites of cleavage and integration of the adaptors, showing the 9bp overhang. Sequenced inserts resulting from one Tn5 cleavage event or independent Tn5 cleavages at the same location map to two different positions separated by 8bp depending on whether they align to the + or - strands. The same Tn5 transposition event is counted at two different locations if unmodified left- or right-most mapping positions are used without modification. **B.** Schematic of two different strategies for "centering" the Tn5 cut position. Shifting the + and - strands by +4 and -5 bp respectively [3] does not align the positions correctly, while shifting by +4/-4 bp results in proper centering. **C.** The pattern of Tn5 cuts in CRM 7, pooled over all samples, resulting from the two strategies of shifting a cut's position to the center of the 9bp overhang.

Fig. S5. Comparison of different strategies for correcting Tn5 DNA sequence bias. The pattern of Tn5 cuts in CRM 7 in one replicate of the 96h IL3+OHT time point. From top to bottom, bias from Tn5 DNA sequence preferences was either not corrected (Uncorrected) or corrected using HINT-ATAC [5], BagFoot [1], or our method (See *Materials and Methods*).

Fig. S6. Enhancer-level accessibility profiles of the *Cebpa* promoter and CRM 16. A,B. Pearson correlation

coefficients of Tn5 counts in each regulatory element, averaged with a 10bp sliding window, computed between each pair of samples. **C,D.** Pooled single-nucleotide accessibility profiles. Black bars are the sum of Tn5 counts over all the samples. Red line is a 3bp sliding-window average. Shaded regions show empirically verified known binding sites [2, 4, 6]. **E.** Scaled ChIP-Seq signal of C/EBPβ in PUER cells (GSM538010). **F.** Scaled ChIP-seq signal of PU.1 (GSM538004) in PUER cells. **A,C,E.** *Cebpa* promoter. **B,D,F.** CRM 16.

Fig. S7. Single nucleotide-resolution dynamics of CRM 16 accessibility during differentiation. The panels show Tn5 cuts at each nucleotide of CRM 16 at different time points and conditions. Differentiation in macrophage (IL3) and neutrophil (GCSF) conditions is shown in green and blue respectively. Replicates have been pooled and normalized by library size.

Fig. S8. Nucleotides in previously validated TF binding sites have fewer Tn5 cuts than those lying outside binding sites. The number of Tn5 cuts at individual nucleotides of an enhancer in each sample is plotted as points. The relative density of the Tn5 cut distribution is shown along the x-axis as a violin plot. Nucleotides lying within previously validated binding sites (Fig. 2 and S6) are plotted separately from those lying outside binding sites; TRUE vs. FALSE respectively. p-values from the one-tailed Wilcoxon rank-sum test, with the alternative that Tn5 distribution of validated binding sites is shifted higher relative to those outside, are shown above the brackets. **A.** *Cebpa* promoter. **B.** CRM 16. **C.** CRM 18. **D.** CRM 7.

Fig. S9. Novel candidate binding sites in CRM 7. Pooled single-nucleotide accessibility profiles. Black bars are the sum of Tn5 counts over all the samples. Shaded regions are footprints that match the binding motifs of the indicated TFs (See *Materials and Methods*).

Fig. S10. Novel candidate binding sites in CRM 18. Pooled single-nucleotide accessibility profiles. Black bars are the sum of Tn5 counts over all the samples. Shaded regions are footprints that match the binding motifs of the indicated TFs (See *Materials and Methods*).

Fig. S11. Novel candidate binding sites in the *Cebpa* promoter. Pooled single-nucleotide accessibility profiles. Black bars are the sum of Tn5 counts over all the samples. Shaded regions are footprints that match the binding motifs of the indicated TFs (See *Materials and Methods*).

Fig. S12. Novel candidate binding sites in CRM 16. Pooled single-nucleotide accessibility profiles. Black bars are the sum of Tn5 counts over all the samples. Shaded regions are footprints that match the binding motifs of the indicated TFs (See *Materials and Methods*).

Fig. S13. The strategy to measure DNA accessibility while distinguishing between the enhancer copies in the endogenous and ROSA26 loci. Reporter cell lines contain two copies of the enhancer (CRM 7 or 18; green), one in the endogenous locus (chromosome 7; red) and the other in the ROSA26 locus (chromosome 6; purple). Tn5 tagmentation during ATAC-Seq library preparation results in DNA fragments having Nextera adaptors at each end (blue and gold line segments). The fragments from the endogenous copy of the enhancer are selectively amplified using a primer complementary to the chromosome 7 DNA sequence neighboring the enhancer (red) and the Nextera index 1 primer (blue). The fragments from the ROSA26 copy of the enhancer are selectively amplified using a primer complementary to the pGL4.17[*luc2/Neo*] DNA sequence neighboring the enhancer in the ROSA26 locus (purple) and the Nextera index 1 primer (blue). The number of such fragments is quantified using qPCR to measure accessibility in a location. A similar scheme is used to measure the accessibility of the endogenous *Cebpa* promoter (not shown) as an internal loading control.

Fig. S14. Enhancer copies in the ROSA26 locus have the same accessibility dynamics as the endogenous ones during neutrophil differentiation. A. The difference in the quantification cycle (ΔC_q) of each enhancer copy and the loading control, the endogenous *Cebpa* promoter. In the boxplots, the horizontal line indicates median over 6 replicate (2 biological $\times$ 3 technical) measurements, the bottom and top edges are the first and third quartiles, while the vertical lines indicate 1.5 times the interquartile range. Values outside this range are shown as points. p-values are from the one-tailed Wilcoxon rank-sum test. **B.** The difference in the quantification cycle (ΔC_q) of the ROSA26 and endogenous copies of each enhancer, normalized by the value at -48 hrs. **C.** Median fold change ($2^{-\Delta C_q}$) in the accessibility of each enhancer copy during neutrophil differentiation. Fold change was scaled to its value at the -48h timepoint.

Fig S15. Single nucleotide-resolution dynamics of CRM 7 accessibility during differentiation. The panels show Tn5 cuts at each nucleotide of CRM 7 at different time points and conditions. Differentiation in macrophage (IL3) and neutrophil (GCSF) conditions is shown in green and blue respectively. Replicates have been pooled and normalized by library size. The 72bp region bordering the Gfi1 site and containing the C/EBP sites is highlighted with the star.

Fig. S16. Single nucleotide-resolution dynamics of CRM 18 accessibility during differentiation. The panels show Tn5 cuts at each nucleotide of CRM 18 at different time points and conditions. Differentiation in macrophage (IL3) and neutrophil (GCSF) conditions is shown in green and blue respectively. Replicates have been pooled and normalized by library size. The 30 bp region bordering the PU.1 and C/EBP sites is highlighted with the star.

Fig. S17. Comparison of accessibility dynamics of key TF binding sites with the rest of the enhancer. The temporal change in average accessibility per nucleotide is plotted for key binding sites (red) and for the rest of the enhancer (blue). **A,C.** CRM 7. The red curve is the change in average accessibility over a 72bp region next to the Gfi1 site and spanning C/EBP sites (highlighted in Fig. S15). **B,D.** CRM 18. The red curve is the change in average accessibility over a 30bp region spanning PU.1 and C/EBP sites (highlighted in Fig. S16). **A,B.** Differentiation in neutrophil conditions (GCSF). **C,D.** Differentiation in macrophage conditions (IL3). Blue shaded region shows period of GCSF pre-treatment prior to induction with OHT during neutrophil differentiation.

Fig. S18. The accessibility of nucleotides neighboring PU.1 binding sites increases with PU.1 occupancy. Genome-wide aggregate PU.1 footprint analysis in undifferentiated PUER cells and 96 hrs after OHT treatment in GCSF conditions. Cut-probability (accessibility) of nucleotides $\pm$100bp from the *Spi1* (PU.1) motif (JASPAR, 2018; MA0080.3). Signal was measured at sites having PWM scores in the top 5%. Undifferentiated PUER cells lack active PU.1 protein and the occupancy of its sites is low. The average accessibility of nucleotides adjacent to the motif is low. OHT treatment activates the PU.1-ER fusion protein, causing it to bind to its sites. The average accessibility of motif-adjacent nucleotides increases significantly.

Fig. S19. Single nucleotide-resolution dynamics of *Cebpa* promoter accessibility during differentiation. The panels show Tn5 cuts at each nucleotide of the *Cebpa* promoter at different time points and conditions. Differentiation in macrophage (IL3) and neutrophil (GCSF) conditions is shown in green and blue respectively. Replicates have been pooled and normalized by library size.

Fig. S20. PU.1 binding in the vicinity of CRM 7. Pooled single-nucleotide accessibility profile of a $\pm$500bp region surrounding CRM 7 in the upper panel. Black bars are the sum of Tn5 counts over all the samples. Shaded regions show empirically verified known binding sites. Scaled ChIP-Seq signal of C/EBPβ (GSM538010) and PU.1 (GSM538004) in PUER cells are shown in the bottom panel. ChIP signal has been normalized to its maxi-

mum in the depicted region.

Fig. S21. Single-nucleotide accessibility of regions containing validated binding sites in CRMs 7 and 18. Black bars are pooled Tn5 cuts at single nucleotides. Shaded regions show validated TF binding sites. **A.** CRM 7 **B-D.** CRM 18.

S2 Supplementary Tables

Notation	Sequence
out-1	5’-CGTCTCGTCGCTGATTGGCTTCTT-3’
out-2	5’-GGAGAGAGGCATTCATGGGAGTG-3’
in-1	5’-CGGCTGTGGGTAGGAGTTAGAT-3
in-2	5’-ATCGCCTTCTACCGCTTGCTCGA-3’
p-1	5’-GTACAAAATACGTGACGTAGAAAG-3’
p-2	5’-TTATTTAACTTGCTATTTCTAGCTCTAAAACTCGTGATCTGCAACTCCAGTCGGTGTTTCGTCCTTTCCACAAGATATATA-3’
p-3	5’-TCAATATTATTGAAGCATTTATCAGGGTTACTAGTACTGGAGTTGCAGATCACGAGGGAGGGAGGGTCAGCGAAAGT-3’
p-4	5’-CCTTAATATTACTTACTTATCCTTGAGAGACGTACTAGTCGAGGGAAGAGGGGGAAG-3’
p-5	5’-TGTGTTGGTTTTTTGTGTGTTCGAACTAGATGCTGTCGACTGATCTGCAACTCCAGTCTTTC-3’
p-6	5’-GAAGGCTCTCAAGGGCATCGGTCGACACTGGAGTTGCAGATCACGAGGGCTGTCTGGTTTCATGAGTCATC-3’
p-7	5’-CCGGTACCTGAGCTCGCTAGCCTCGAGGATATCAGAATTCCCATGGAGATCTAACTCCTACCCACAGCCG-3’
p-8	5’-ATTCGCGACCCGAAGCTGAAGCTTCCATGGGAATTCGGCAATCCGGTACTGTTGGTAAAGCCACCATGGA-3’
p-9	5’-AGTAAAACCTCTACAAATGTGGTAAAATCGATAAGAATTCCCATGGGGATCCCCCACTTCCACCCCCTAAGA-3’
p-10	5’-TGTTAAGGTTGCTCTGCTCAGGGGATCCCCATGGGAATTCGTTTGCGTATTGGGCGCTCTTCCGCTGATCTGCG-3’
p-11	5’-TCTTTATTTTCATTACATCTGTGTGTTGGTTTTTTGTGTGGAATTCCCATGGTTCGAAATGCCACCCCTCTGATTTTGC-3’
p-12	5’-TCTATTCCAGGGGACTCAGTGTTCGAACCATGGGAATTCCTAGATGCTGTCGACTGATCTGCAACTCCAGTCTTTCTAG-3’
p-13	5’-TCTTTATTTTCATTACATCTGTGTGTTGGTTTTTTGTGTGGAATTCCCATGGTTCGAAAAAATCAGTTTATCCCTATGCTGCC-3’
p-14	5’-CAGAGTTGTCCTCAGCCCATTCGAACCATGGGAATTCCTAGATGCTGTCGACTGATCTGCAACTCCAGTCTTTCTAG-3’
p-15	5’-AAGAATTCCCATGGGGATCC-3’
p-16	5’-TGGAATTCCCATGGTTCGAA-3’
p-17	5’-TTGAAGCCCACCAAGAGCAC-3’
p-18	5’-AAGCTCTCCTCTTTCCCTCCT-3’
p-19	5’-CCAACTGGCTCGCGC-3’
p-20	5’-CAAGCAGAAGACGGCATACGAGATTCGCCTTAGTCTCGTGGGCTCGG-3’

Table S1

Gene	CRM	Chromosome	Start	End	Tfs
Cebpa	7	7	35127019	35127034	Cebp
Cebpa	7	7	35127036	35127044	Runx1
Cebpa	7	7	35127059	35127096	Cebp, Ikzf1
Cebpa	7	7	35127096	35127116	Cebp
Cebpa	7	7	35127217	35127258	Jun/Fos, Cebp
Cebpa	18	7	35156512	35156526	MZF1, Runx1
Cebpa	18	7	35156578	35156593	Runx1
Cebpa	18	7	35156695	35156709	Jun/Fos
Cebpa	18	7	35156719	35156730	Runx1
Cebpa	18	7	35156782	35156814	Runx1, GATA-3, MZF1, Sp1, Ikzf1
Cebpa	18	7	35156815	35156842	Jun/Fos, Cebp, Runx1
Cebpa	18	7	35156846	35156862	Ikzf1, Runx1
Cebpa	0	7	35118286	35118321	MZF1
Cebpa	0	7	35118321	35118336	Sp1, MZF1
Cebpa	0	7	35118762	35118787	Cebp
Cebpa	0	7	35118787	35118808	Ikzf1
Cebpa	0	7	35118981	35119020	Sp1, MZF1, Runx1
Cebpa	16	7	35150786	35150805	Ikzf1, Sp1
Cebpa	16	7	35150857	35150882	Runx1
Cebpa	16	7	35150899	35150933	Ikzf1, MZF1, Runx1, PU.1
Cebpa	16	7	35151025	35151042	PU.1
Cebpa	16	7	35151147	35151166	Ikzf1, Runx1
Cebpa	16	7	35151335	35151355	Gata3
Cebpa	16	7	35151360	35151381	Ikzf1, Runx1
Cebpa	16	7	35151381	35151411	Jun/Fos
Cebpa	16	7	35151411	35151441	Cebp, MZF1, Ikzf1, Runx1

Table S2

S3 Supplementary Figures

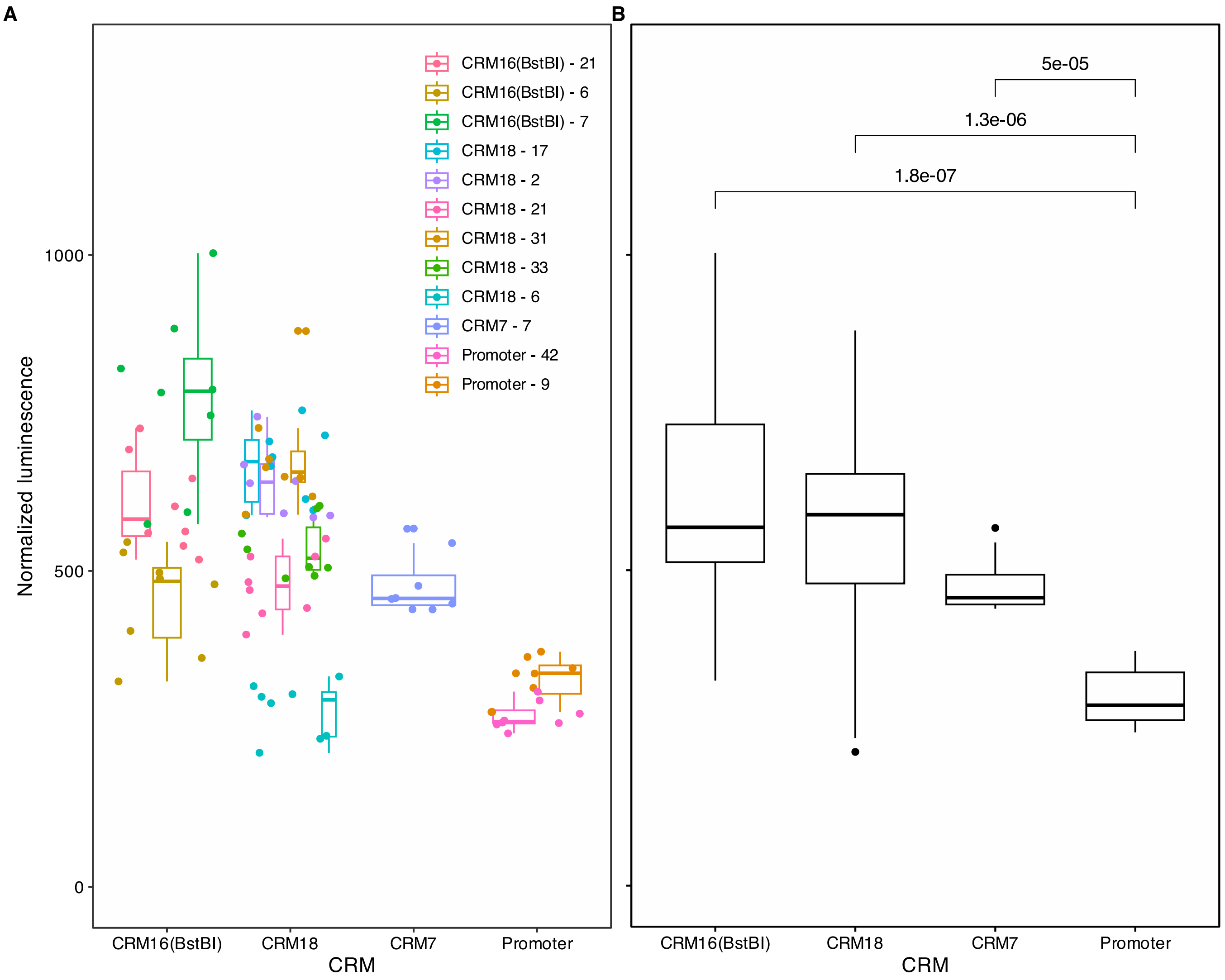

Figure S1

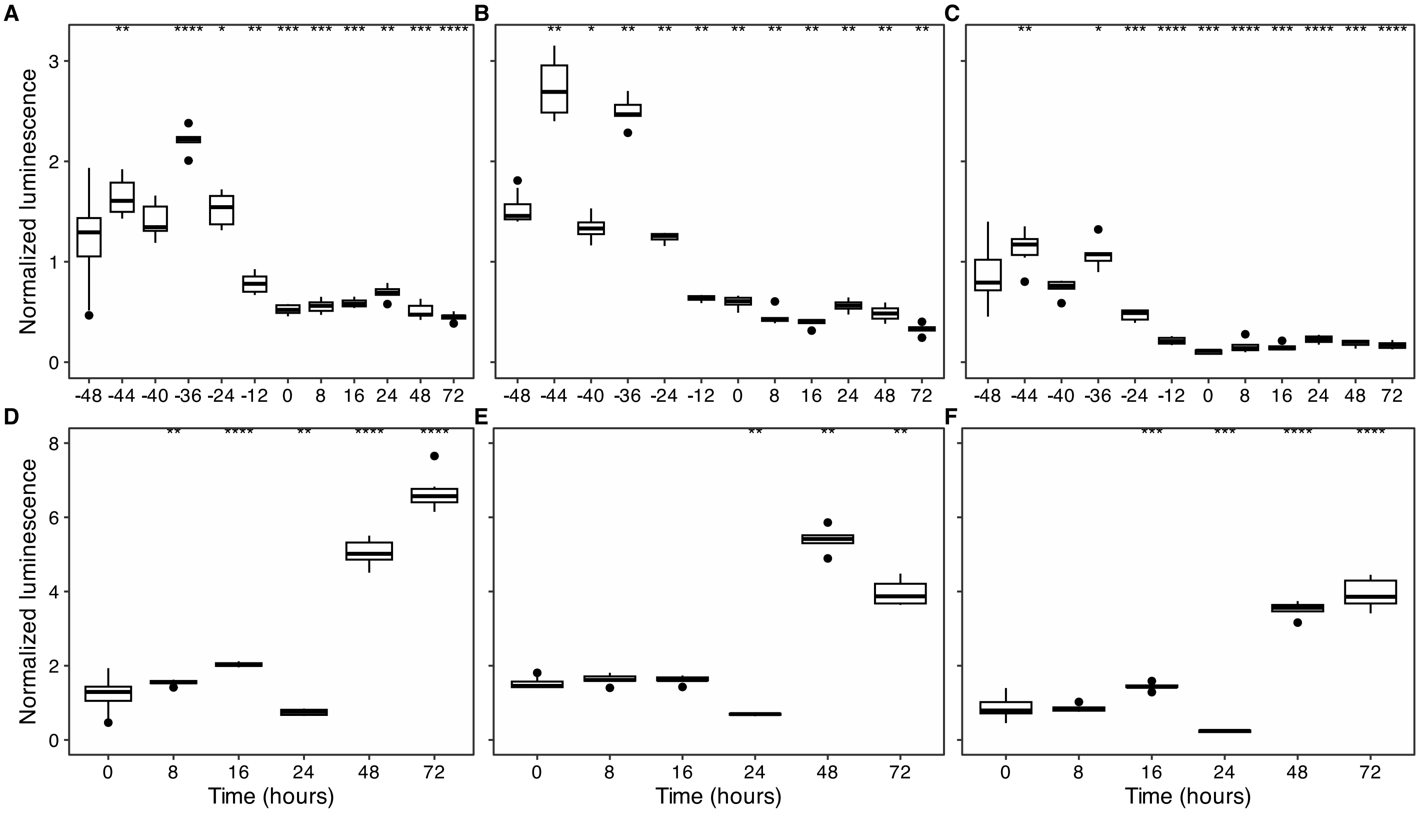

Figure S2

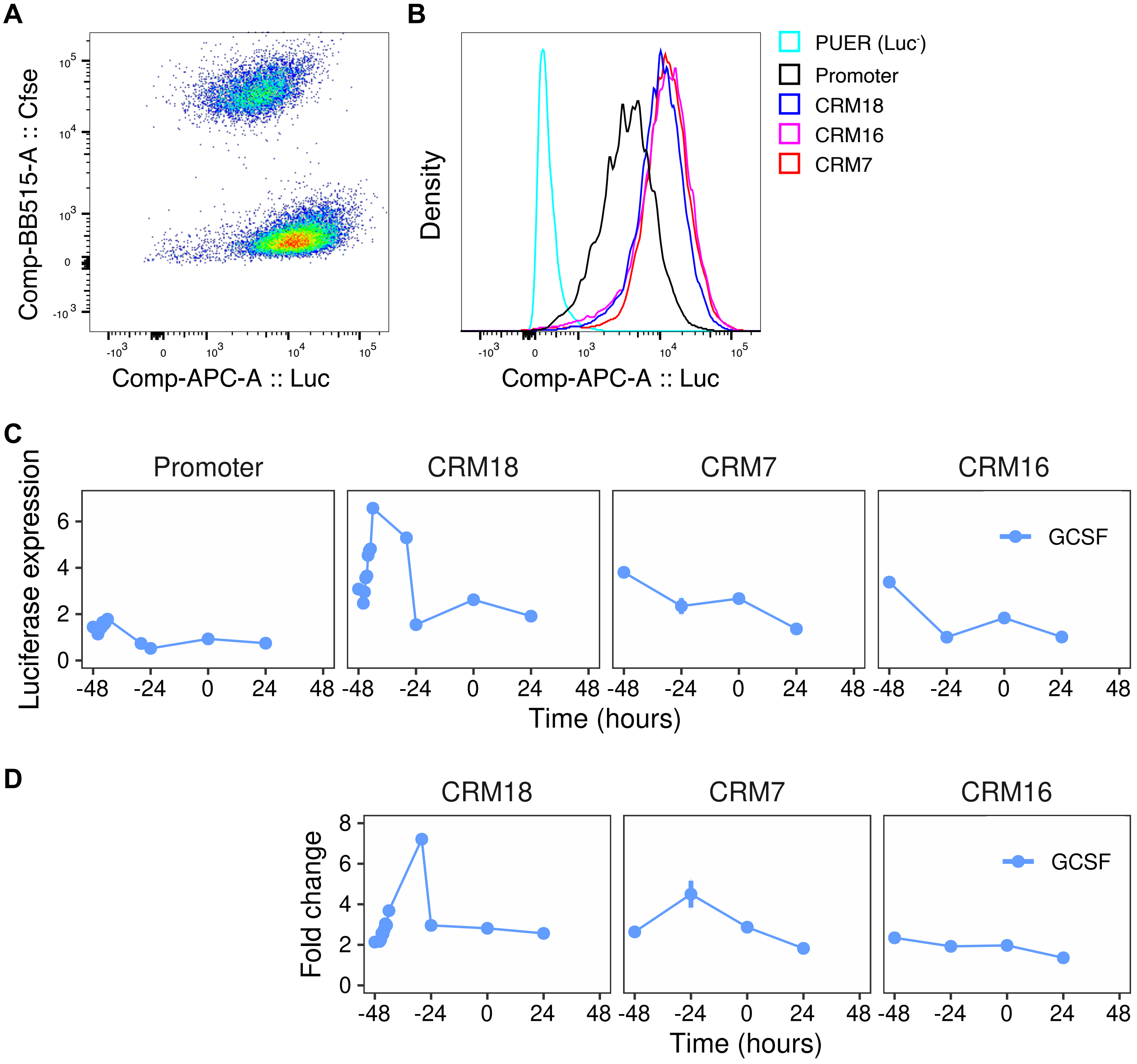
A
B
C
D
Comp-BB515-A :: Cfse
Comp-APC-A :: Luc
Density
PUER (Luc-)
Promoter
CRM18
CRM16
CRM7
Luciferase expression
Promoter
CRM18
CRM7
CRM16
GCSF
Time (hours)
Fold change
CRM18
CRM7
CRM16
GCSF
Time (hours)

Figure S3

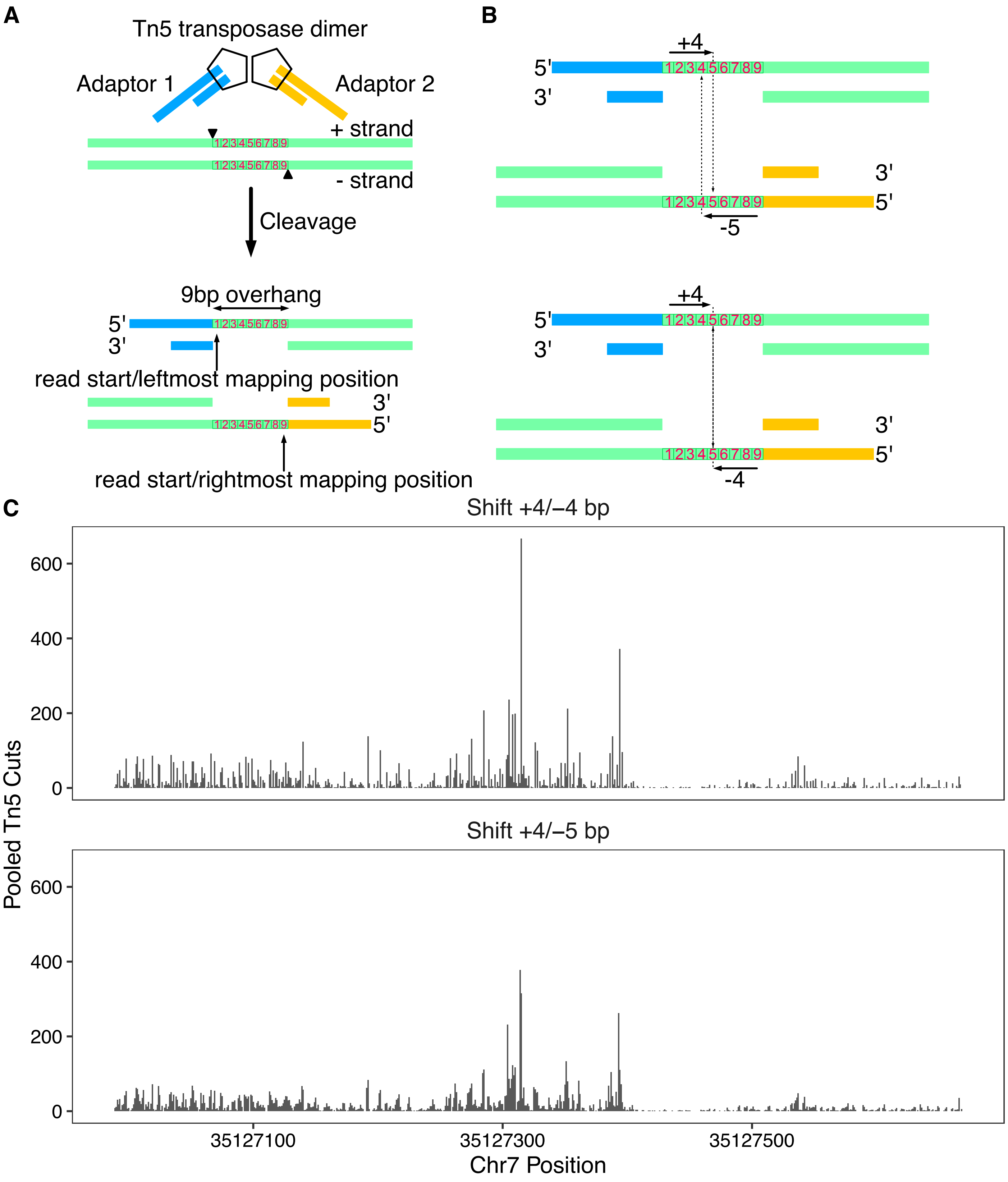
A
Tn5 transposase dimer
Adaptor 1
Adaptor 2
+ strand
- strand
Cleavage
9bp overhang
5'
3'
read start/leftmost mapping position
read start/rightmost mapping position
B
+4
-5
-4
C
Shift +4/–4 bp
Shift +4/–5 bp
Pooled Tn5 Cuts
0
200
400
600
35127100
35127300
35127500
Chr7 Position

Figure S4

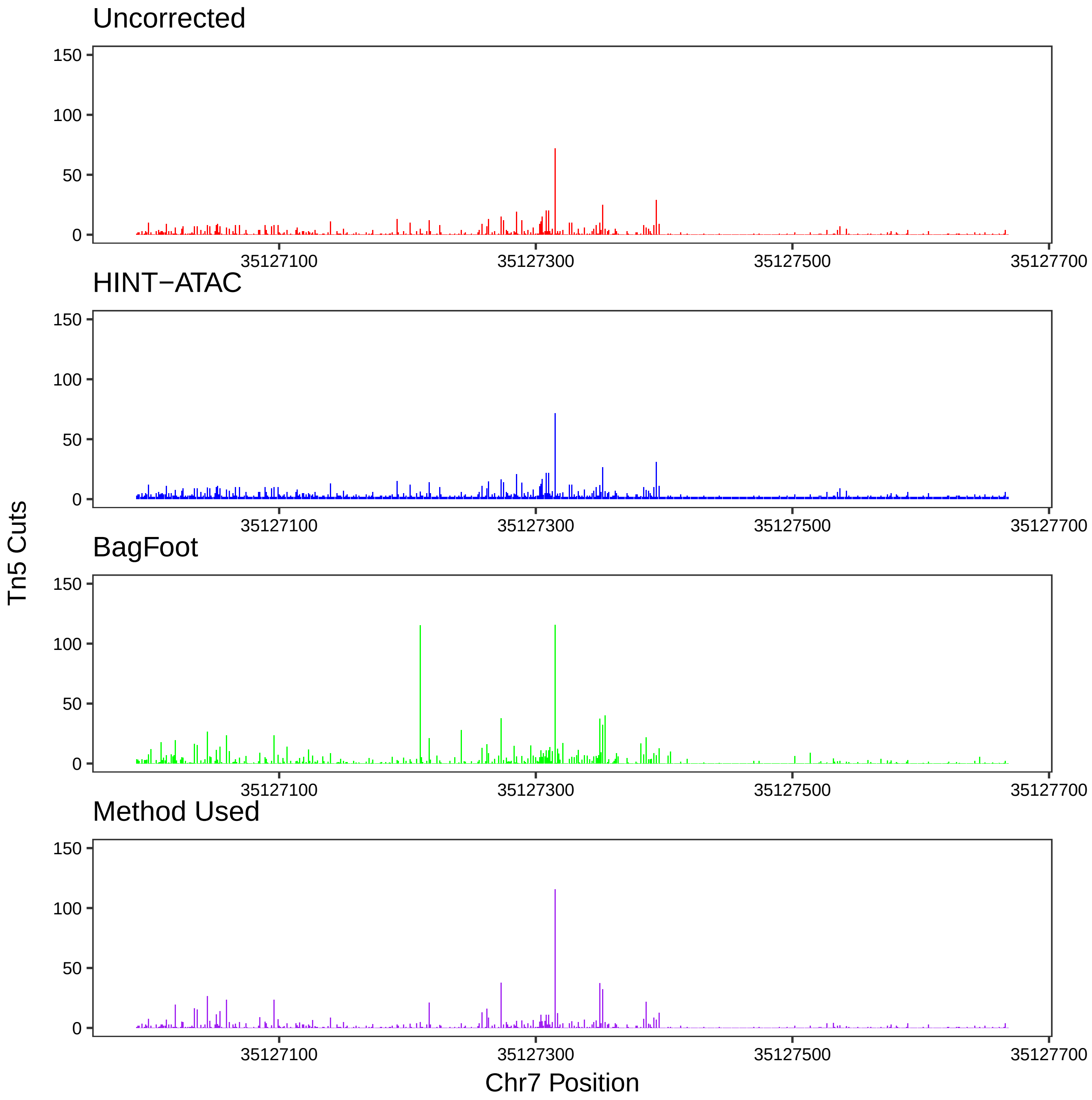
Uncorrected
150
100
50
0
35127100
35127300
35127500
35127700
HINT−ATAC
150
100
50
0
35127100
35127300
35127500
35127700
BagFoot
150
100
50
0
35127100
35127300
35127500
35127700
Method Used
150
100
50
0
35127100
35127300
35127500
35127700
Tn5 Cuts
Chr7 Position

Figure S5

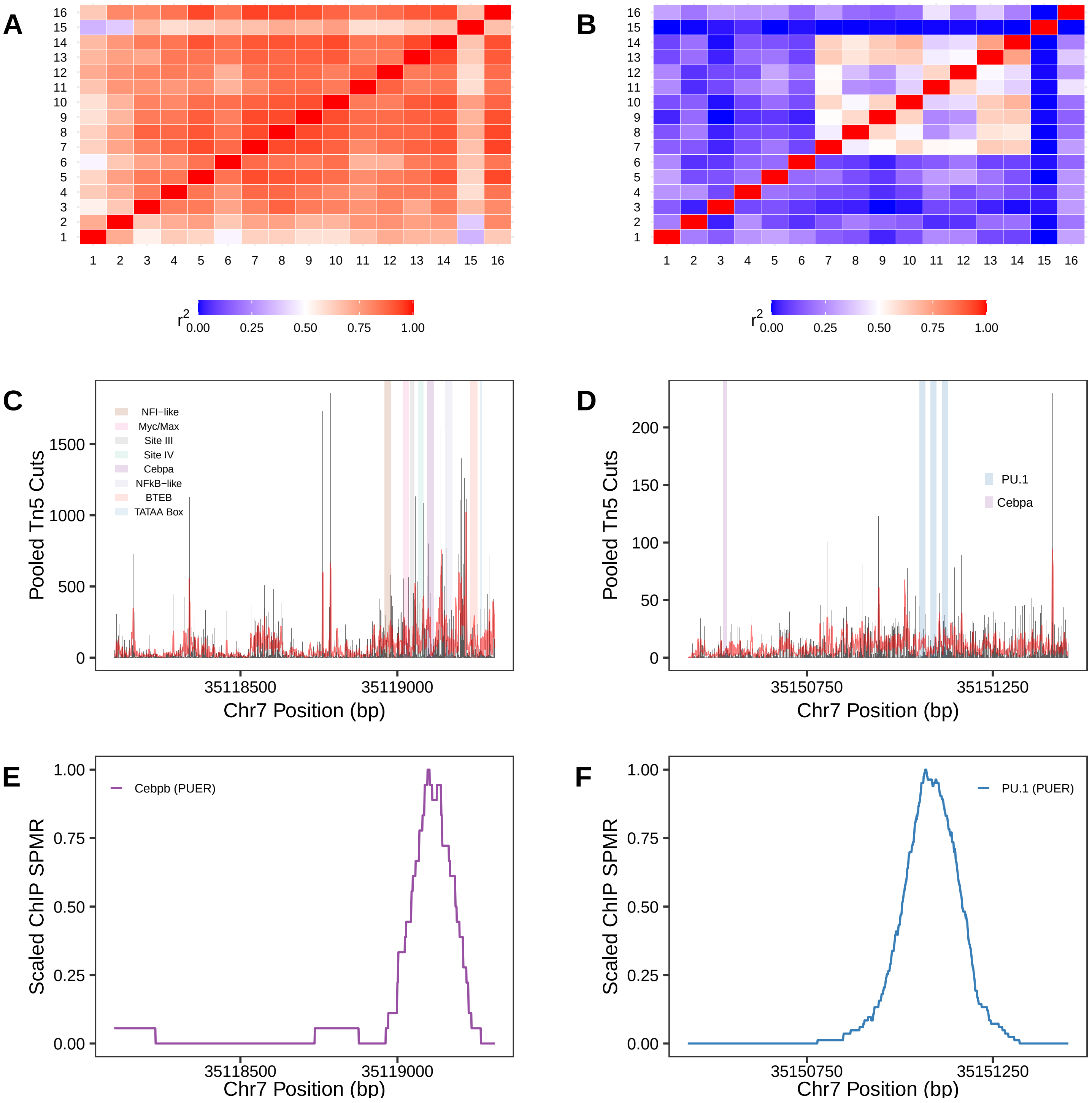
A
B
r2
0.00
0.25
0.50
0.75
1.00
C
NFI−like
Myc/Max
Site III
Site IV
Cebpa
NFkB−like
BTEB
TATAA Box
Pooled Tn5 Cuts
Chr7 Position (bp)
35118500
35119000
D
PU.1
Cebpa
35150750
35151250
E
Cebpb (PUER)
Scaled ChIP SPMR
F
PU.1 (PUER)

Figure S6

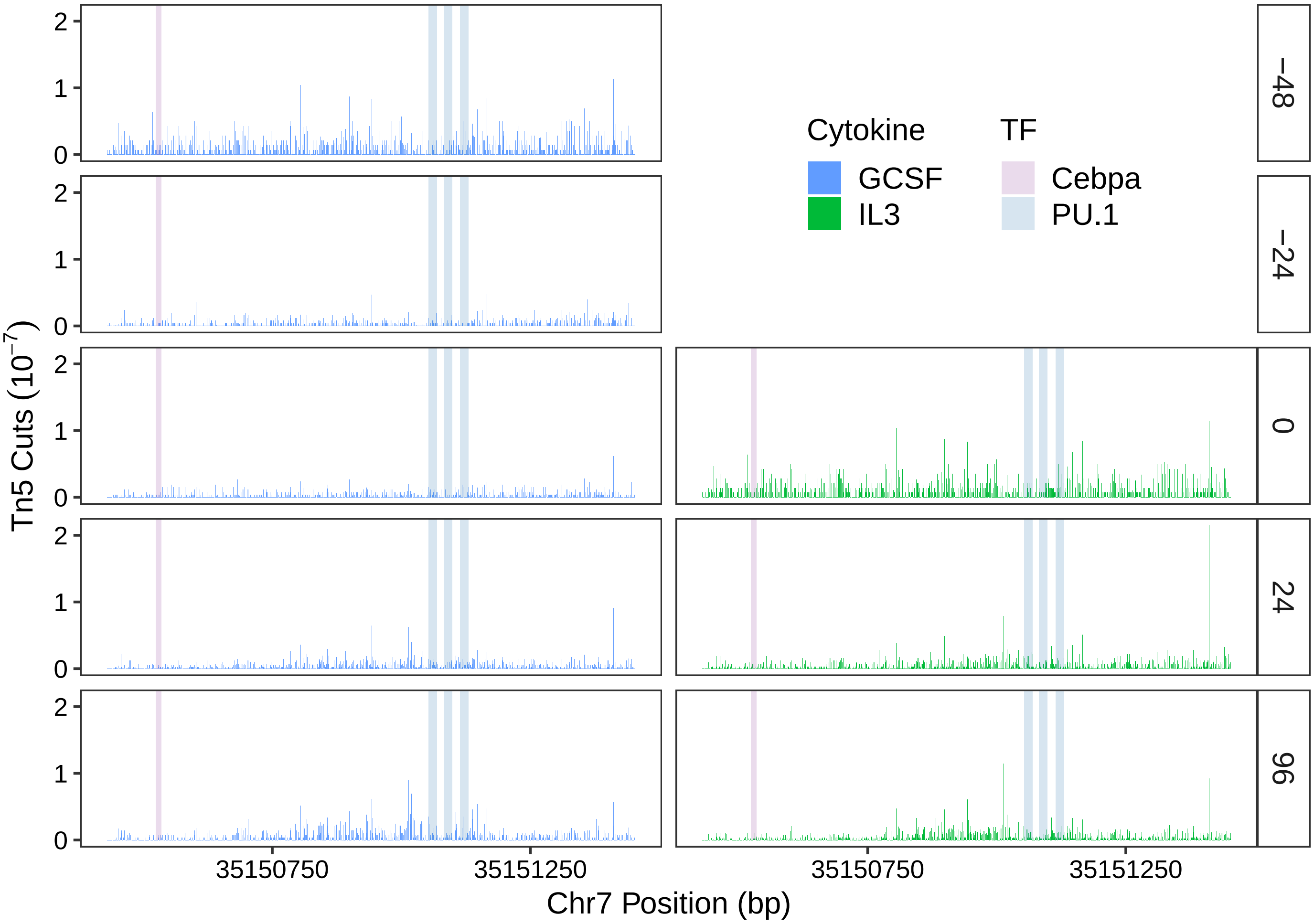

Tn5 Cuts (10^−7)
Cytokine
GCSF
IL3
TF
Cebpa
PU.1
−48
−24
0
24
96
0
1
2
35150750
35151250
Chr7 Position (bp)

Figure S7

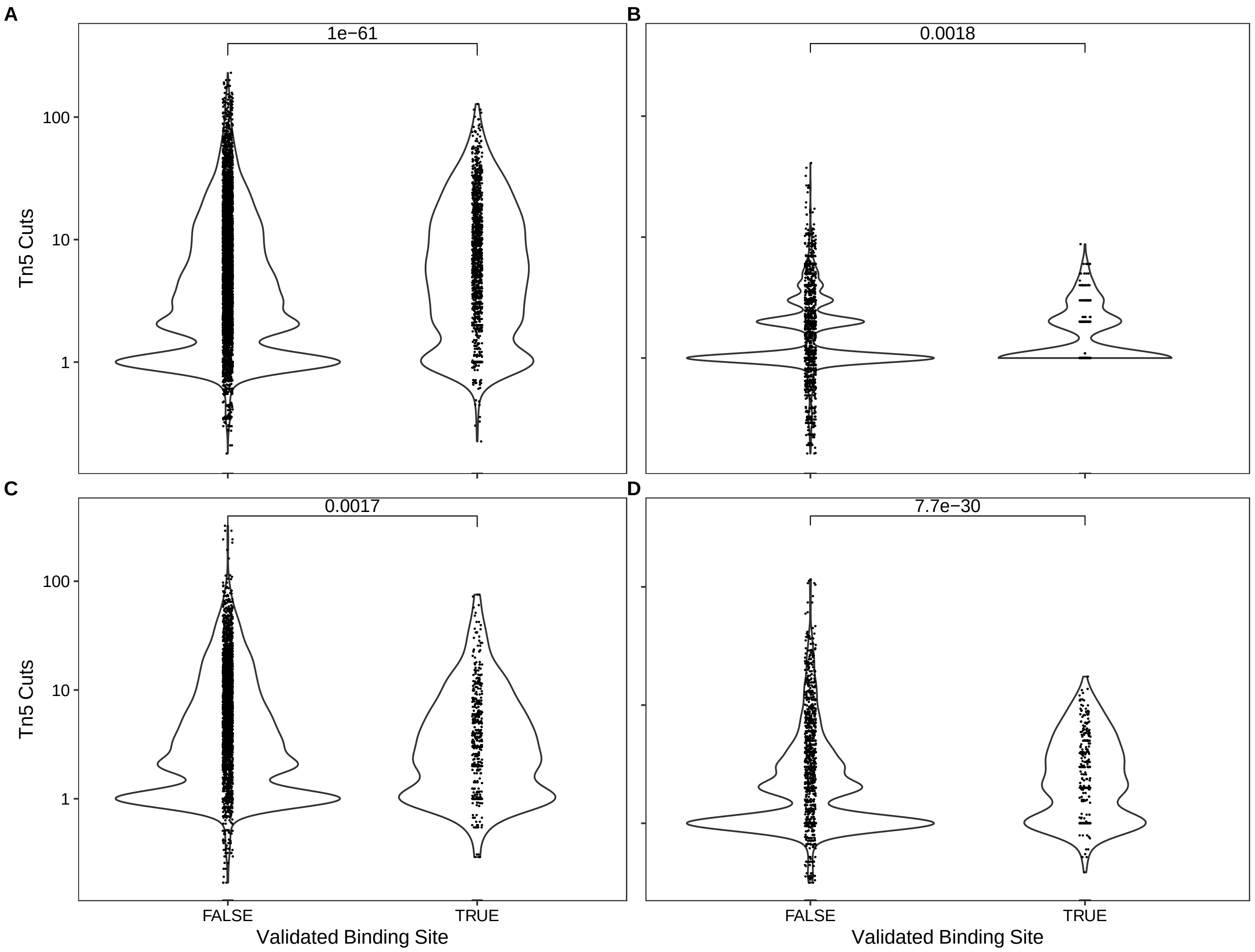
A
B
C
D
1e−61
0.0018
0.0017
7.7e−30
Tn5 Cuts
100
10
1
FALSE
TRUE
Validated Binding Site

Figure S8

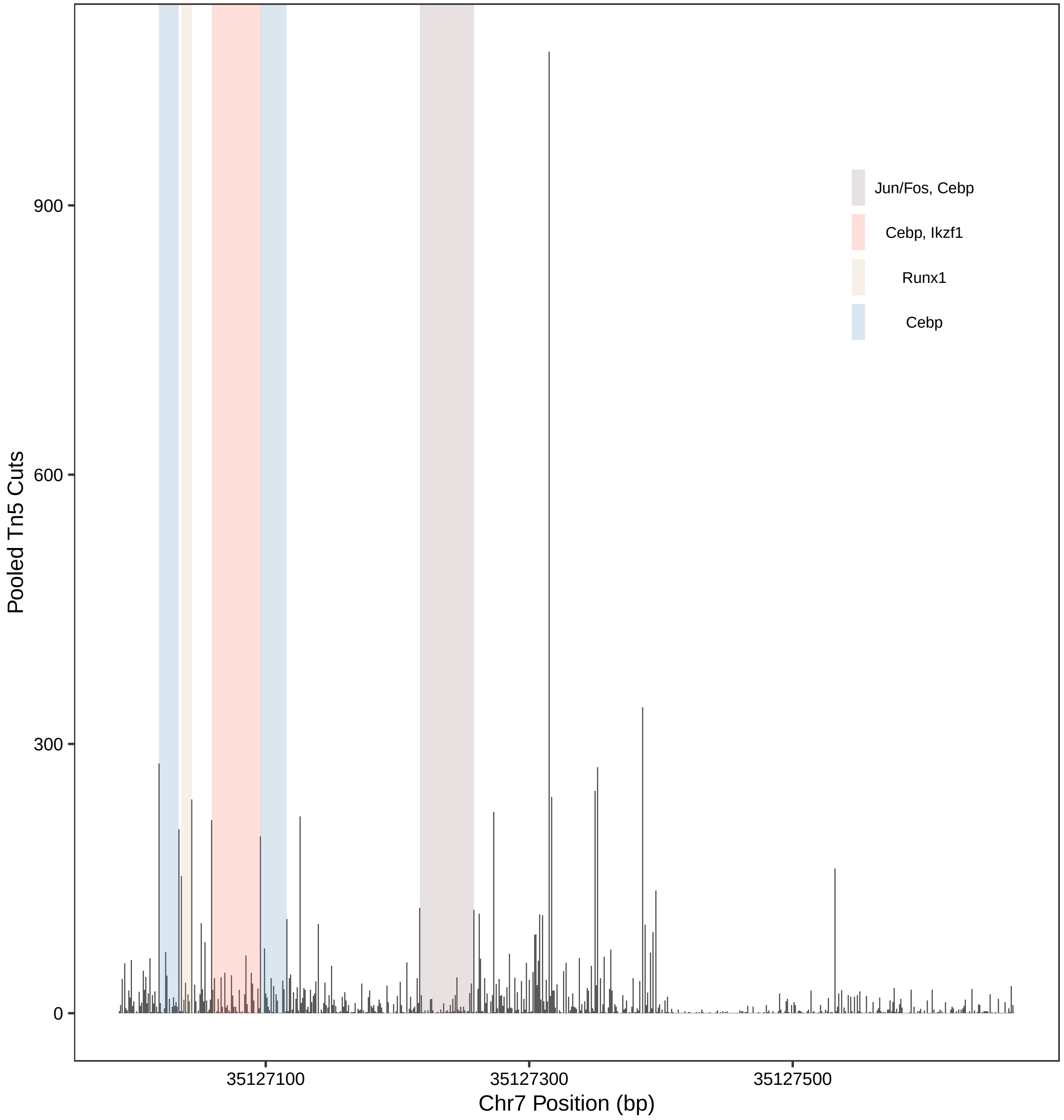
Jun/Fos, Cebp
Cebp, Ikzf1
Runx1
Cebp
Pooled Tn5 Cuts
900
600
300
0
35127100
35127300
35127500
Chr7 Position (bp)

Figure S9

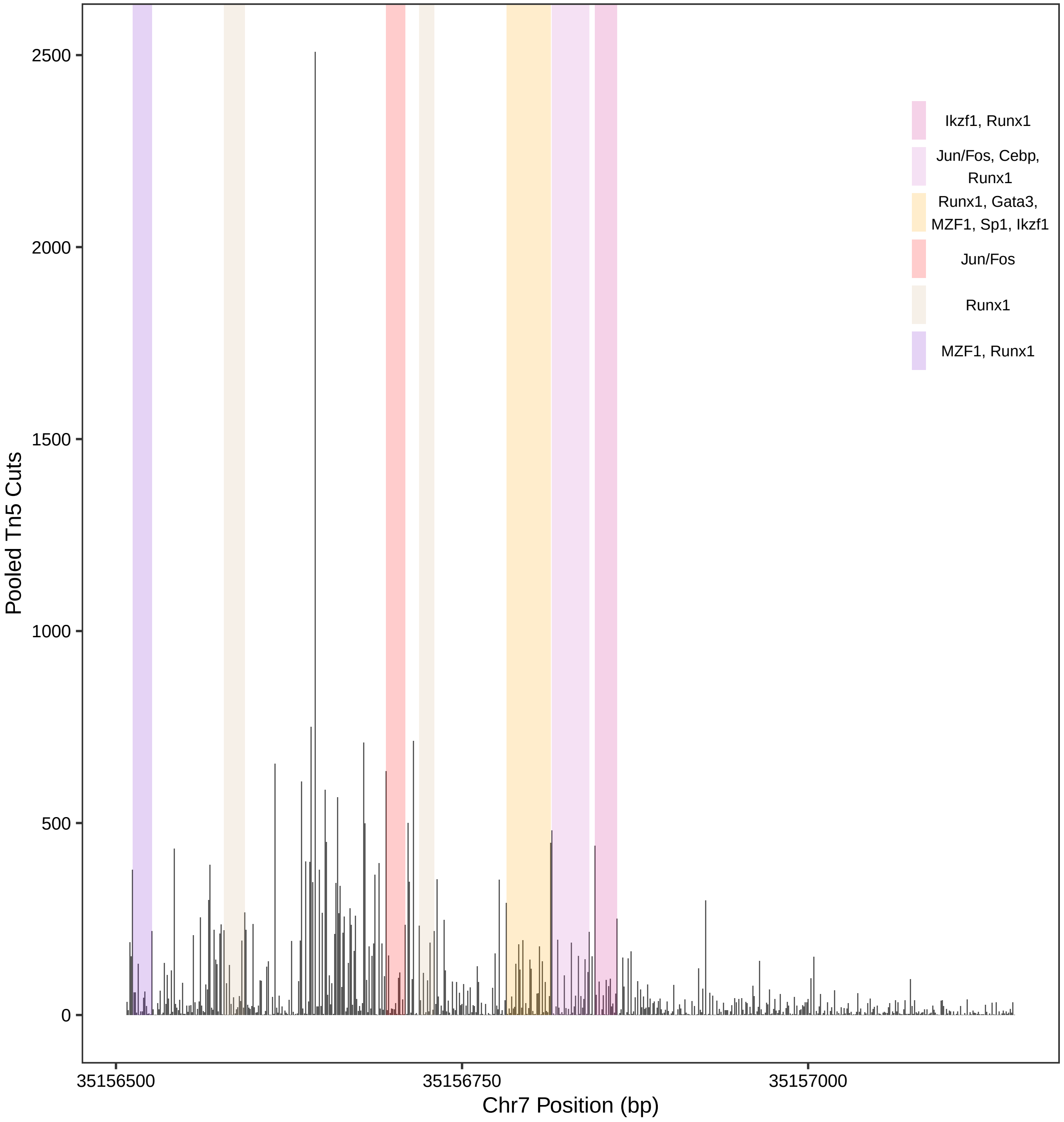
Pooled Tn5 Cuts
2500
2000
1500
1000
500
0
35156500
35156750
35157000
Chr7 Position (bp)
Ikzf1, Runx1
Jun/Fos, Cebp, Runx1
Runx1, Gata3, MZF1, Sp1, Ikzf1
Jun/Fos
Runx1
MZF1, Runx1

Figure S10

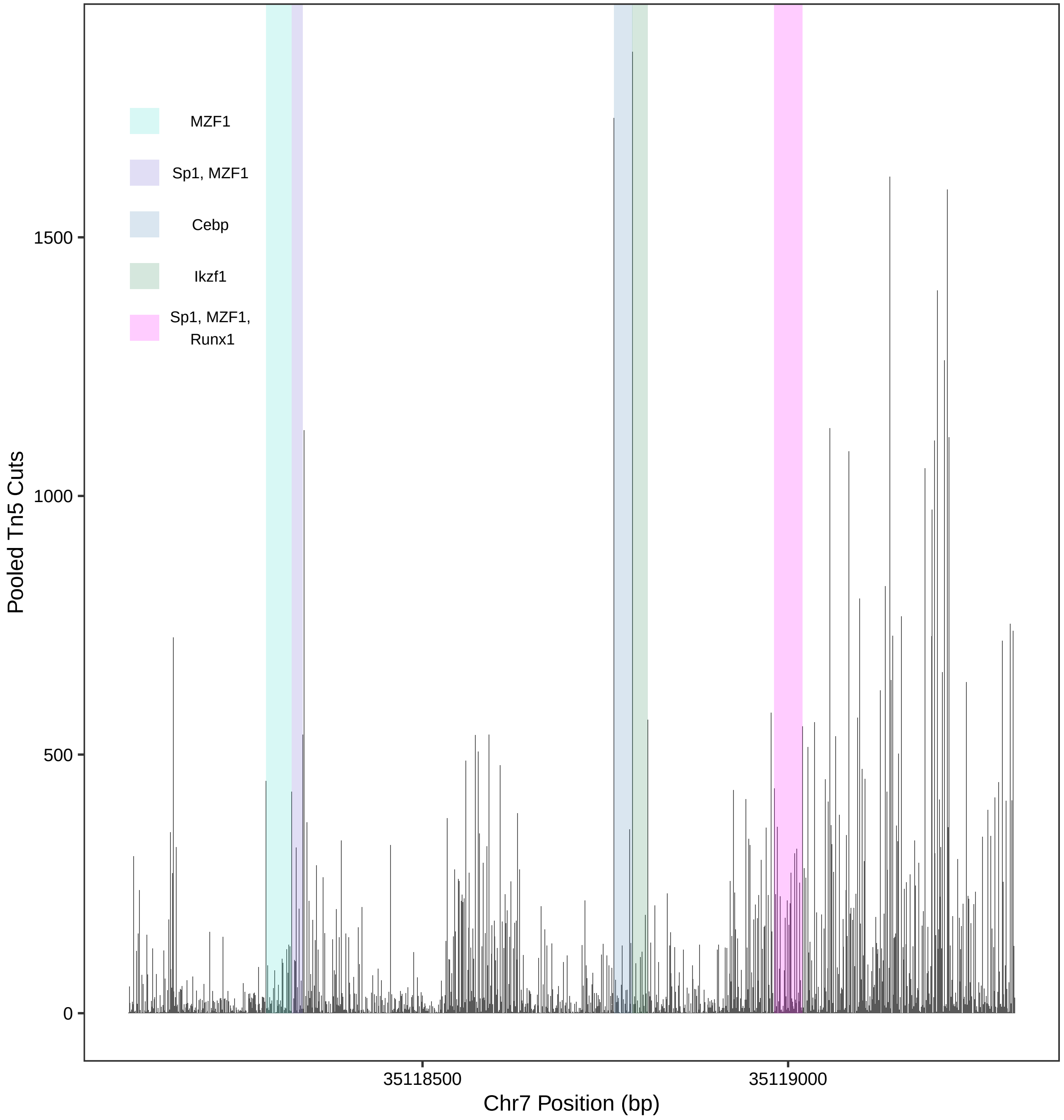
MZF1
Sp1, MZF1
Cebp
Ikzf1
Sp1, MZF1, Runx1
Pooled Tn5 Cuts
1500
1000
500
0
35118500
35119000
Chr7 Position (bp)

Figure S11

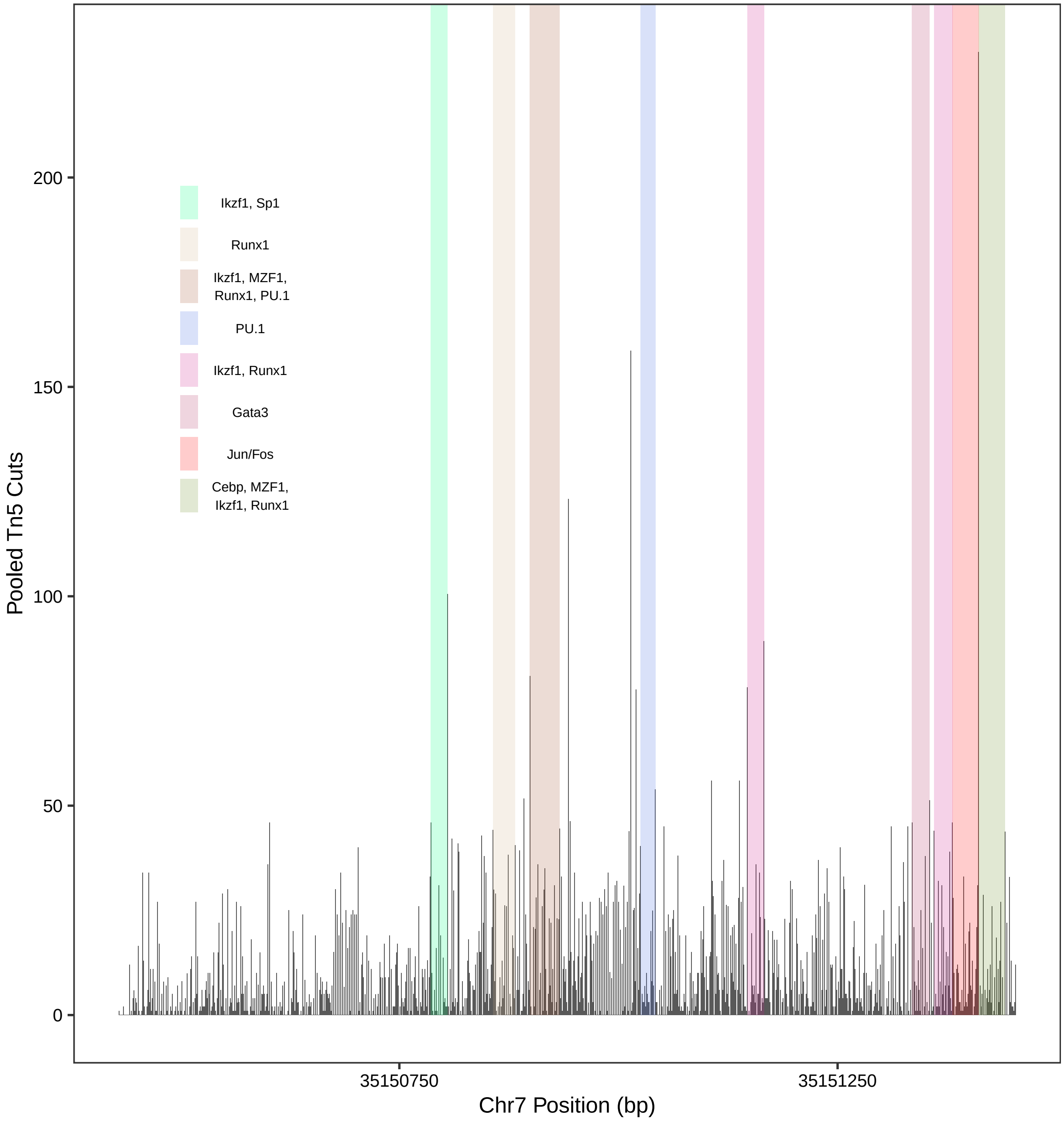
Ikzf1, Sp1
Runx1
Ikzf1, MZF1,
Runx1, PU.1
PU.1
Ikzf1, Runx1
Gata3
Jun/Fos
Cebp, MZF1,
Ikzf1, Runx1
Pooled Tn5 Cuts
0
50
100
150
200
35150750
35151250
Chr7 Position (bp)

Figure S12

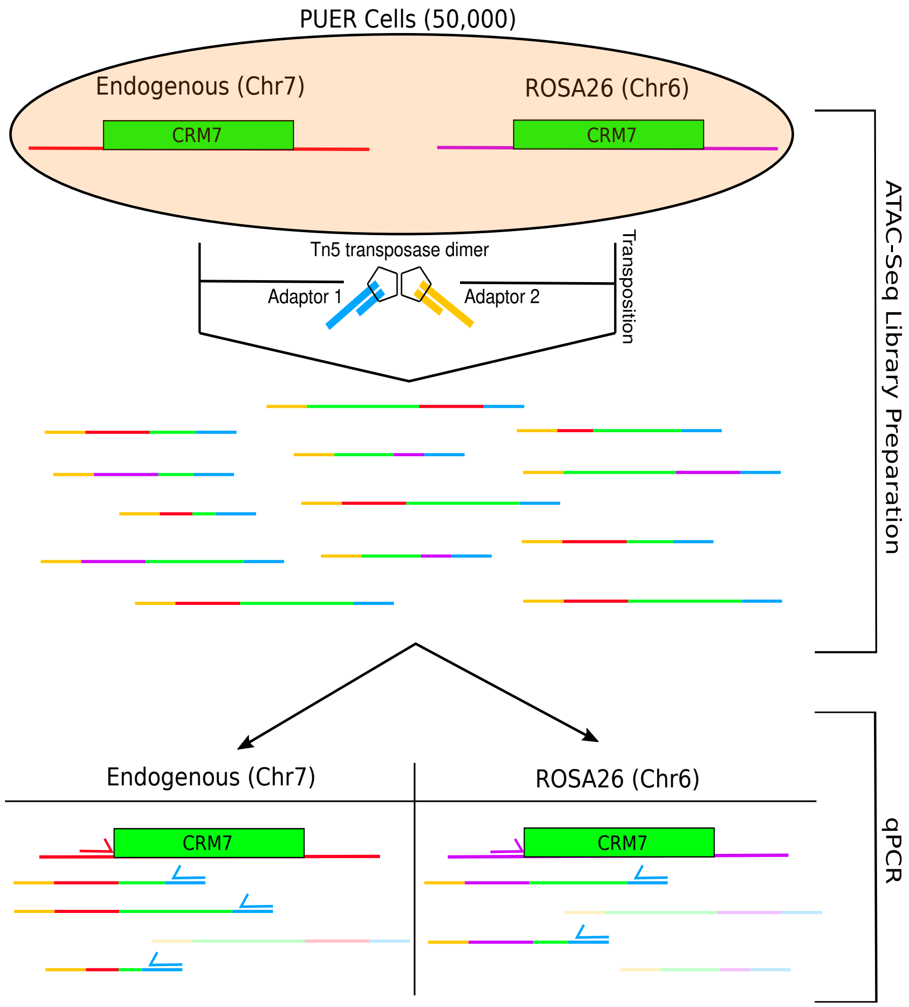

PUER Cells (50,000)
Endogenous (Chr7)
ROSA26 (Chr6)
CRM7
CRM7
Tn5 transposase dimer
Adaptor 1
Adaptor 2
Transposition
ATAC-Seq Library Preparation
Endogenous (Chr7)
ROSA26 (Chr6)
CRM7
CRM7
qPCR

Figure S13

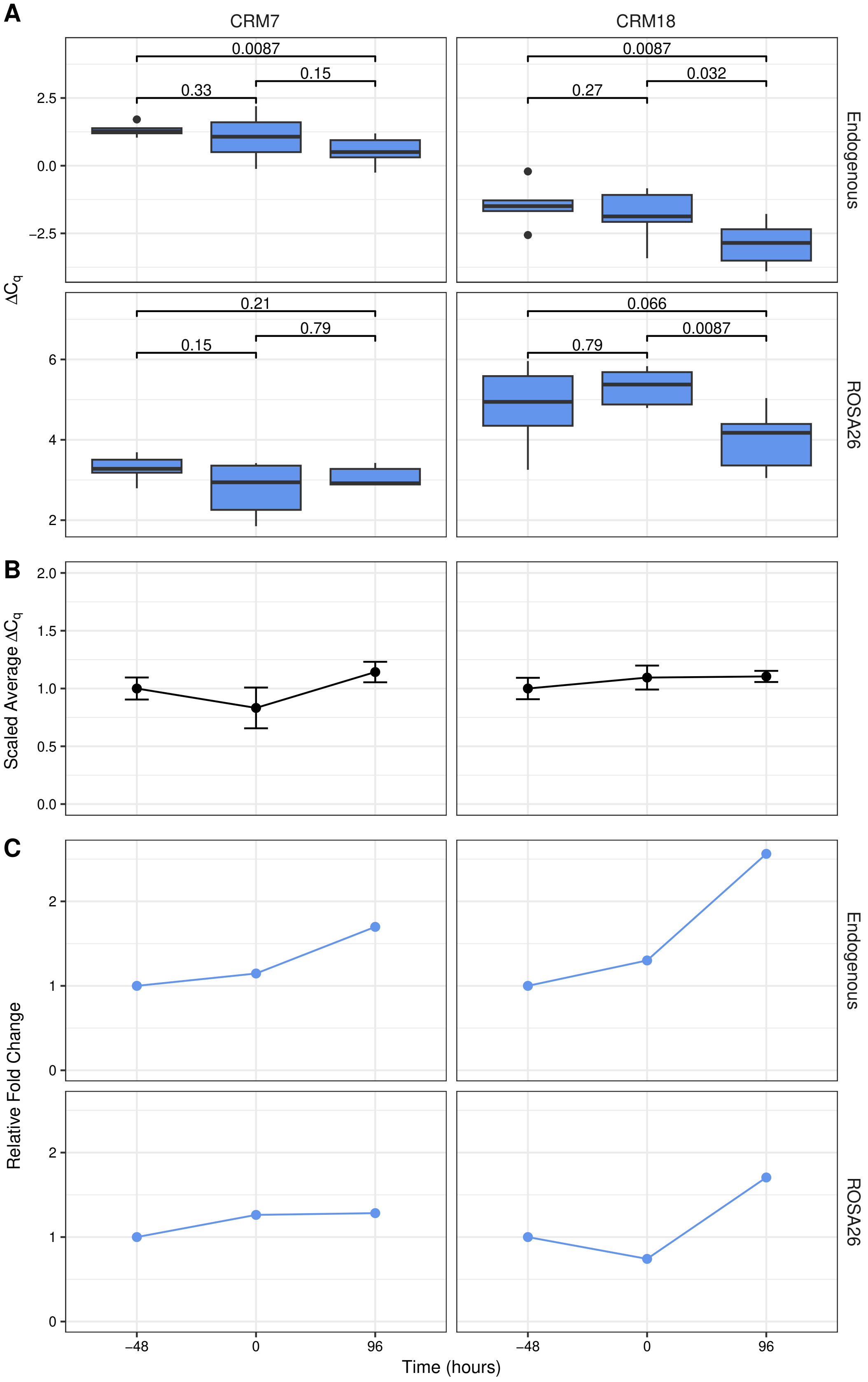
A
CRM7
CRM18
0.0087
0.15
0.33
0.0087
0.032
0.27
2.5
0.0
−2.5
Endogenous
ΔCq
0.21
0.79
0.15
0.066
0.0087
0.79
6
4
2
ROSA26
B
2.0
1.5
1.0
0.5
0.0
Scaled Average ΔCq
C
Relative Fold Change
Endogenous
ROSA26
–48
0
96
Time (hours)

Figure S14

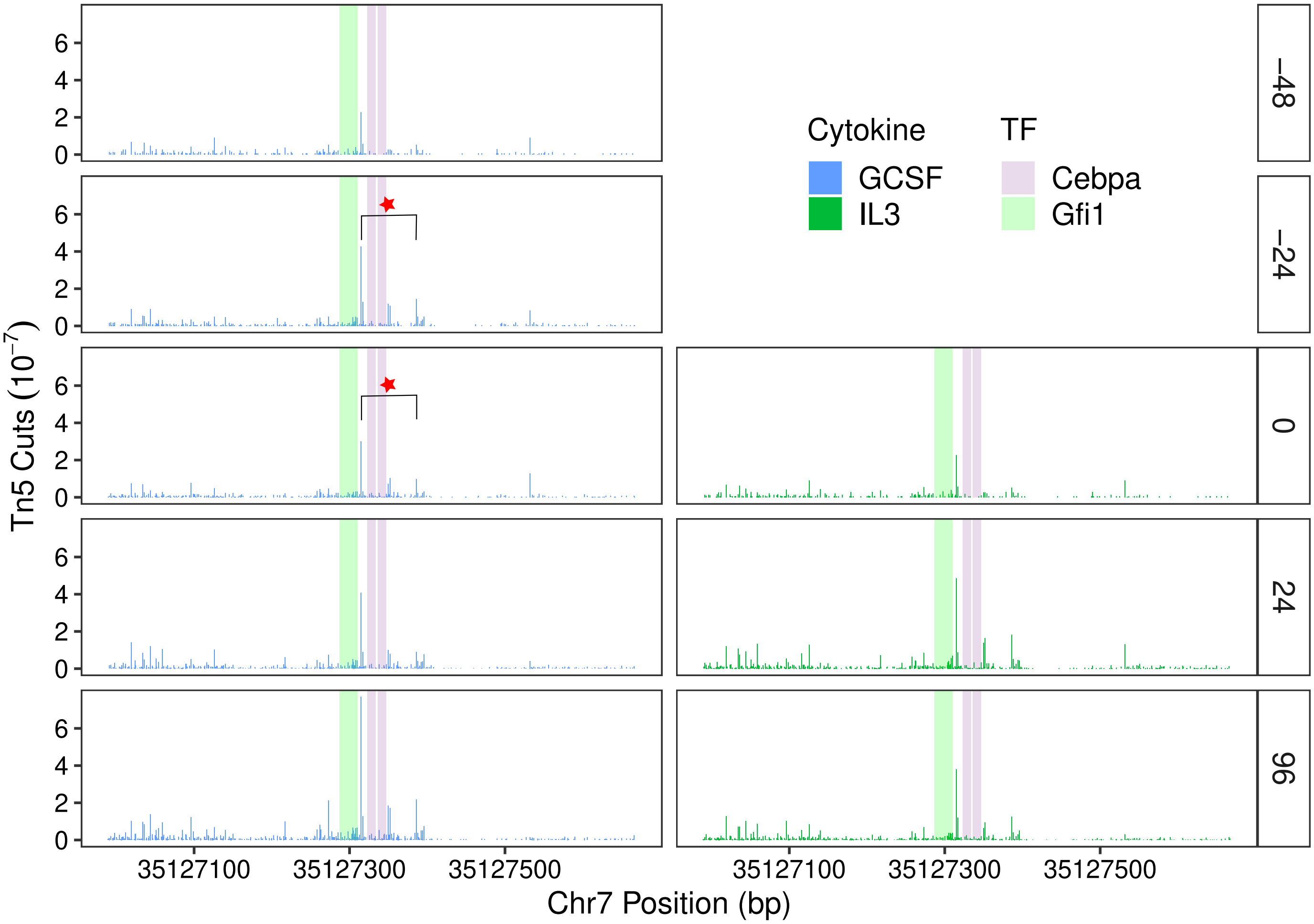

Tn5 Cuts (10^{-7})
Chr7 Position (bp)
35127100
35127300
35127500
0
2
4
6
−48
−24
0
24
96
Cytokine
GCSF
IL3
TF
Cebpa
Gfi1

Figure S15

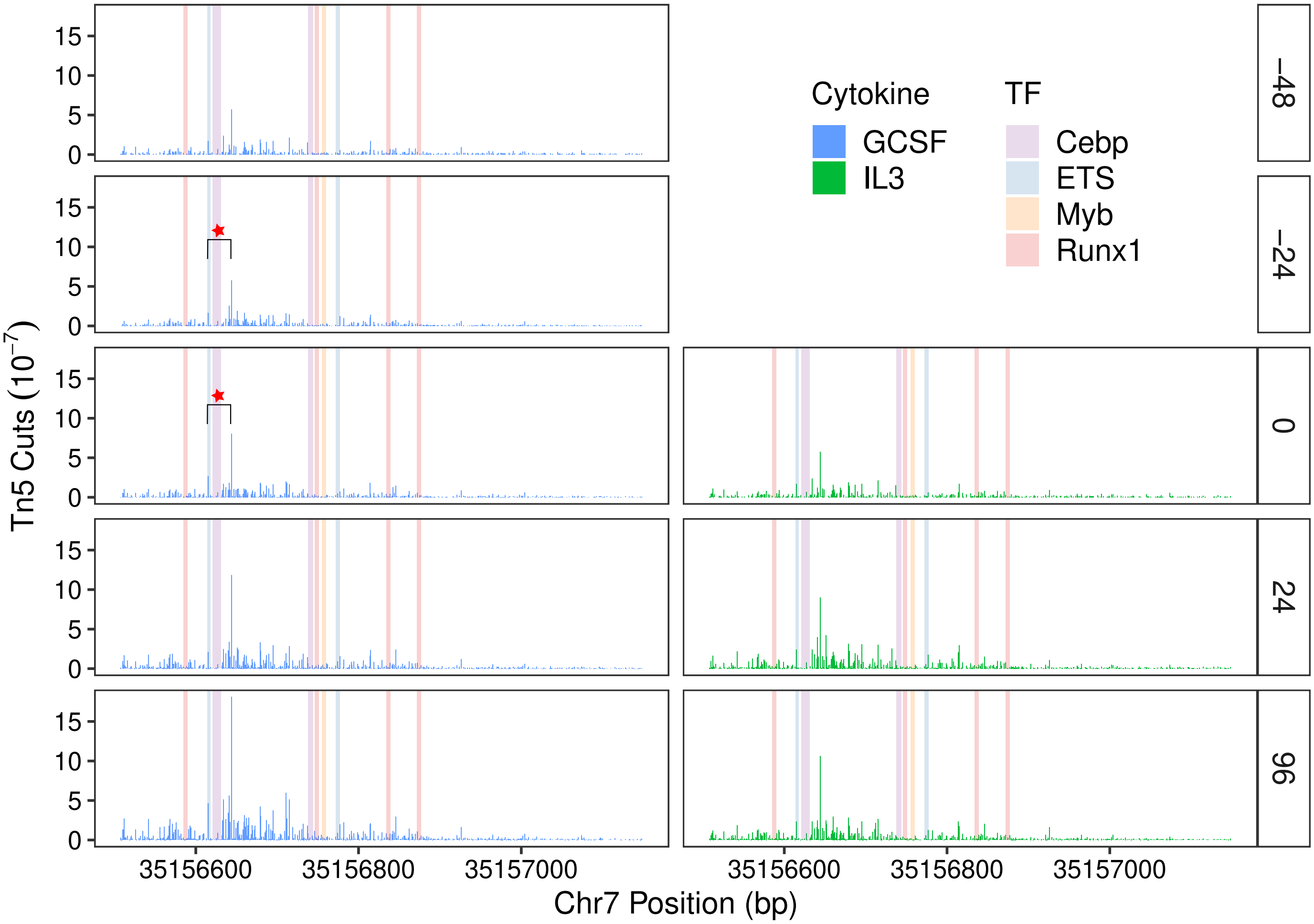

Tn5 Cuts (10^{-7})
15
10
5
0
Cytokine
GCSF
IL3
TF
Cebp
ETS
Myb
Runx1
−48
−24
0
24
96
35156600
35156800
35157000
Chr7 Position (bp)

Figure S16

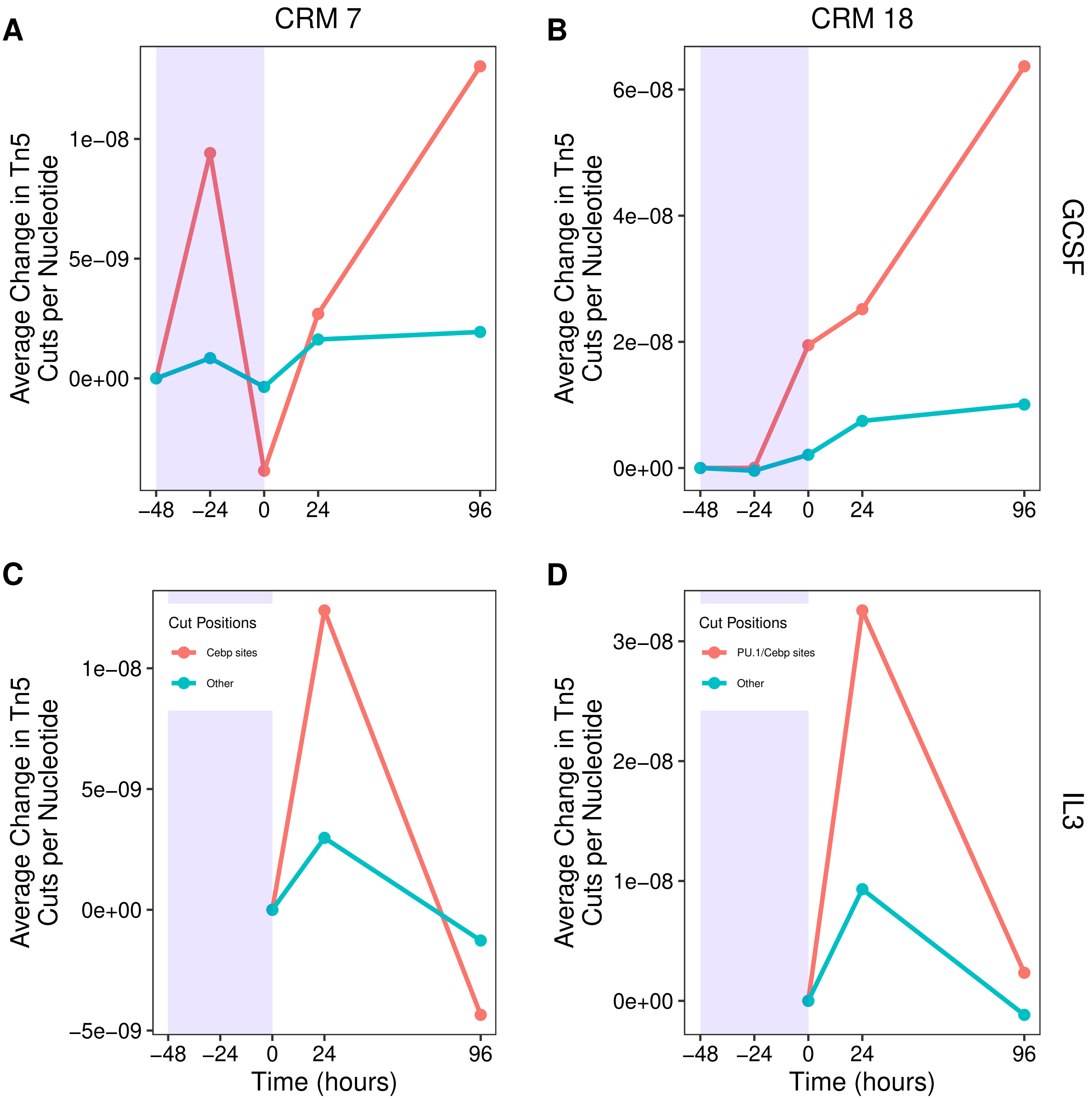
A
CRM 7
B
CRM 18
GCSF
C
D
IL3
Average Change in Tn5 Cuts per Nucleotide
1e−08
5e−09
0e+00
−5e−09
6e−08
4e−08
2e−08
3e−08
−48 −24 0 24 96
Time (hours)
Cut Positions
Cebp sites
PU.1/Cebp sites
Other

Figure S17

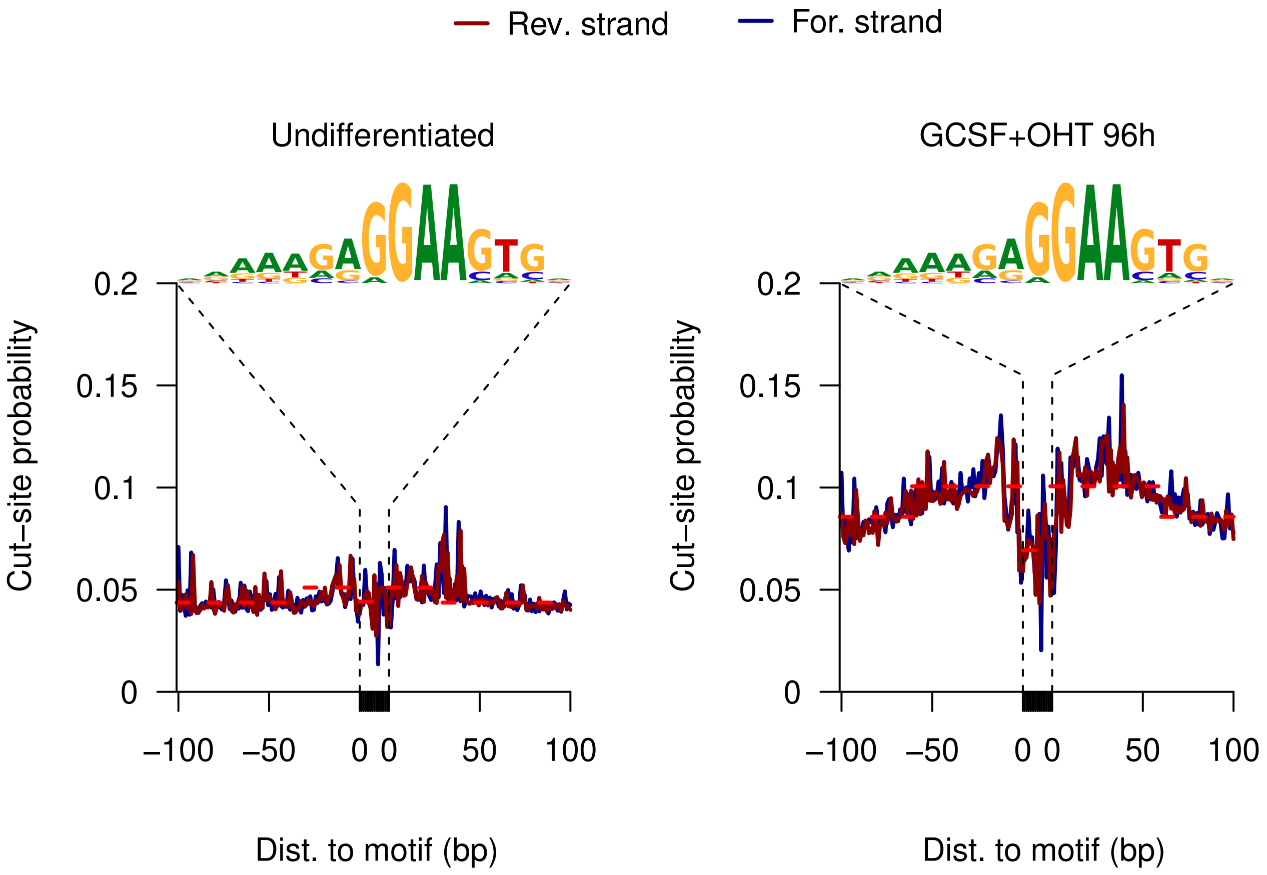

Rev. strand
For. strand
Undifferentiated
GCSF+OHT 96h
Cut−site probability
0.2
0.15
0.1
0.05
0
−100
−50
0 0
50
100
Dist. to motif (bp)

Figure S18

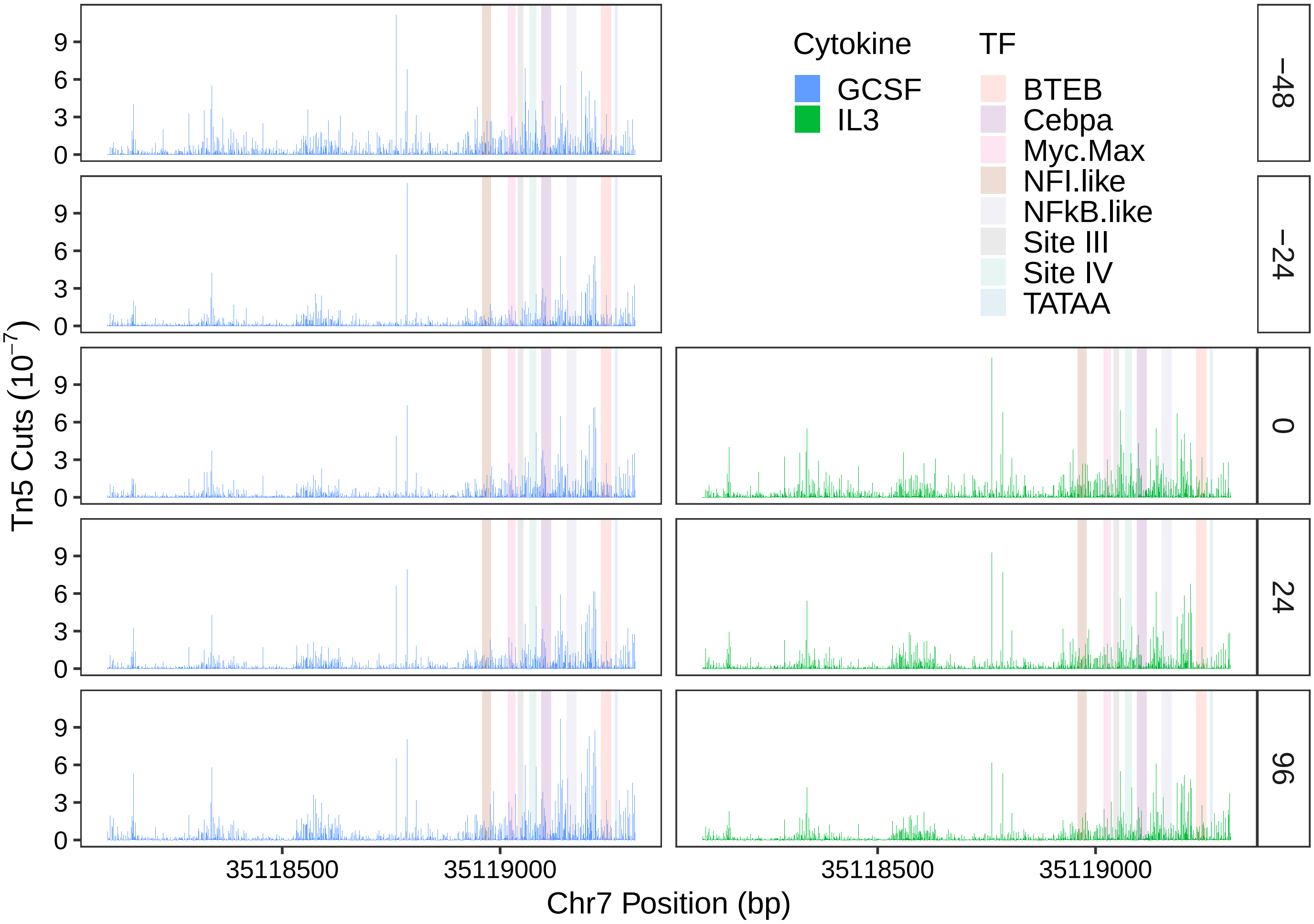
Cytokine
GCSF
IL3
TF
BTEB
Cebpa
Myc.Max
NFI.like
NFkB.like
Site III
Site IV
TATAA
−48
−24
0
24
96
Tn5 Cuts (10^−7)
9
6
3
0
35118500
35119000
Chr7 Position (bp)

Figure S19

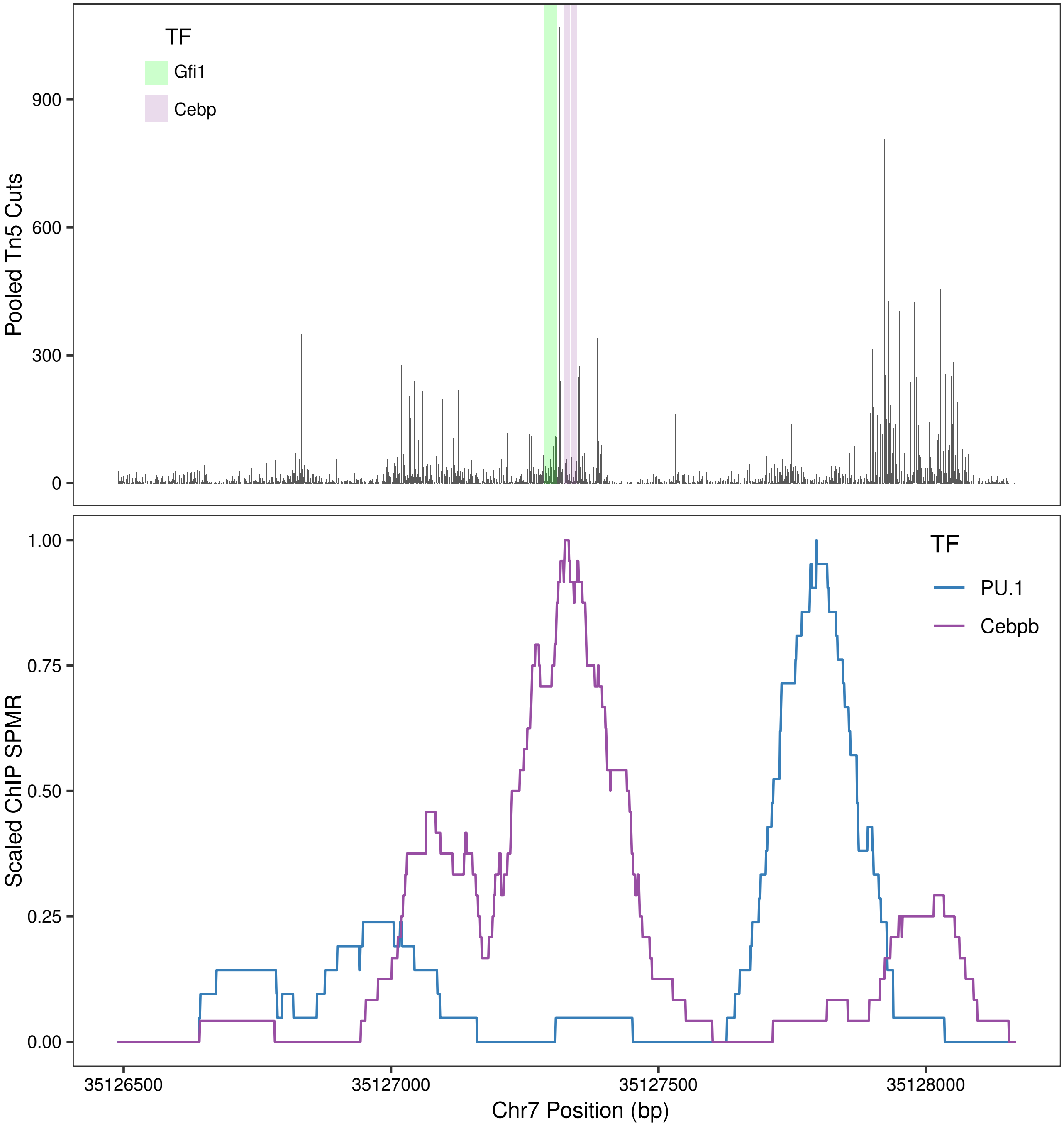

TF
Gfi1
Cebp
Pooled Tn5 Cuts
900
600
300
0
1.00
0.75
0.50
0.25
0.00
Scaled ChIP SPMR
TF
PU.1
Cebpb
35126500
35127000
35127500
35128000
Chr7 Position (bp)

Figure S20

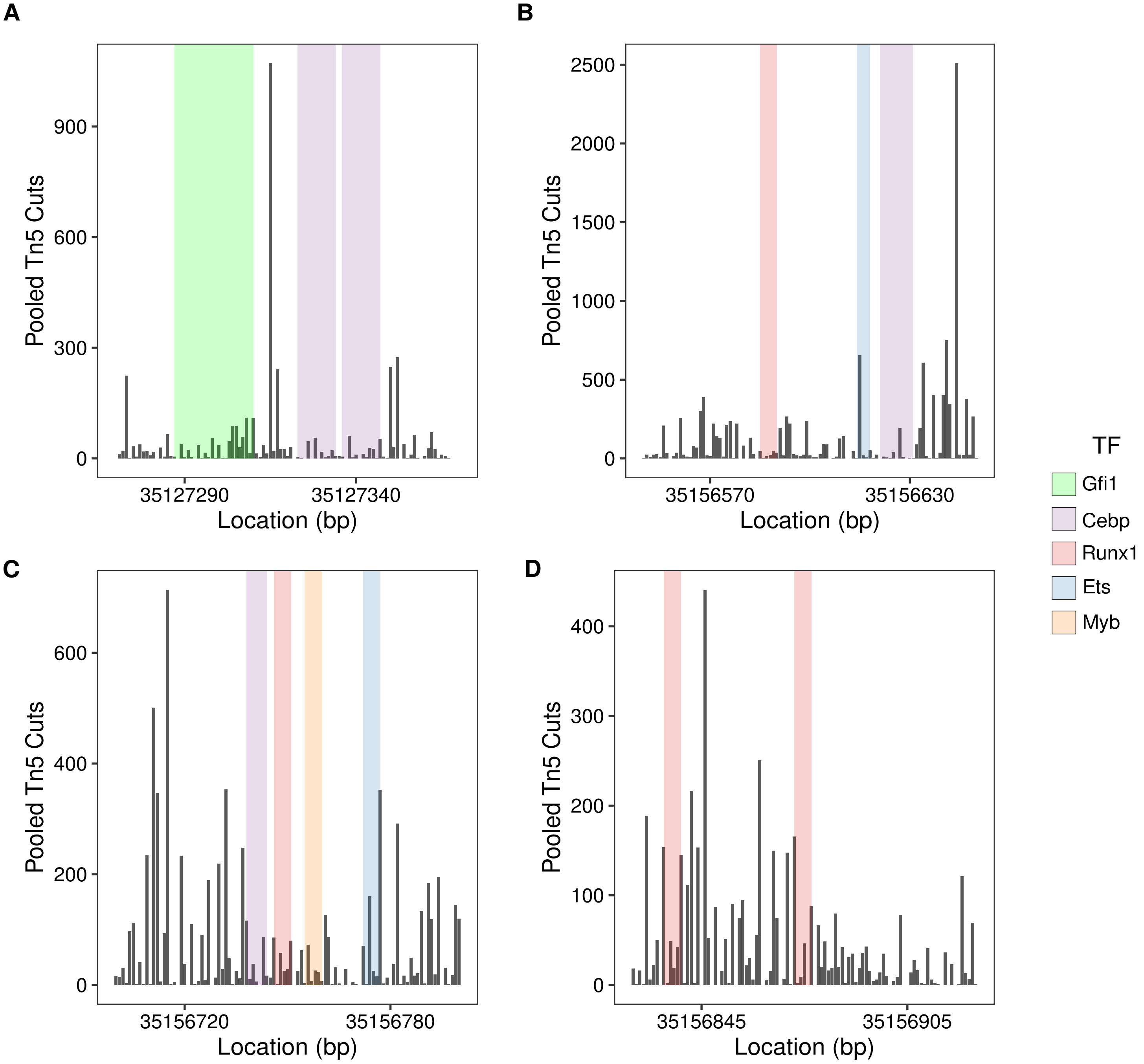
A
B
C
D
Pooled Tn5 Cuts
Location (bp)
0
300
600
900
35127290
35127340
500
1000
1500
2000
2500
35156570
35156630
200
400
600
35156720
35156780
100
200
300
400
35156845
35156905
TF
Gfi1
Cebp
Runx1
Ets
Myb

Figure S21

References

[1] Baek S, Goldstein I, Hager GL. 2017. Bivariate genomic footprinting detects changes in transcription factor activity. Cell Rep. 19:1710–1722.

[2] Bertolino E, Reinitz J, Manu. 2016. The analysis of novel distal cebpa enhancers and silencers using a transcriptional model reveals the complex regulatory logic of hematopoietic lineage specification. Dev Biol. 413:128–44.

[3] Buenrostro JD, Giresi PG, Zaba LC, Chang HY, Greenleaf WJ. 2013. Transposition of native chromatin for fast and sensitive epigenomic profiling of open chromatin, dna-binding proteins and nucleosome position. Nat Methods. 10:1213–8. Cited in PubMed; PMID 24097267.

[4] Legraverend C, Antonson P, Flodby P, Xanthopoulos KG. 1993. High level activity of the mouse ccaat/enhancer binding protein (c/ebp alpha) gene promoter involves autoregulation and several ubiquitous transcription factors. Nucleic Acids Res. 21:1735–42.

[5] Li Z, Schulz MH, Look T, Begemann M, Zenke M, Costa IG. 2019. Identification of transcription factor binding sites using atac-seq. Genome Biol. 20:45.

[6] Repele A, Krueger S, Bhattacharyya T, Tuineau MY, Manu. 2019. The regulatory control of cebpa enhancers and silencers in the myeloid and red-blood cell lineages. PLoS One. 14:e0217580.
